# Multi-omics analysis of patient-derived organoids reveals that E3 ligase COP1 promotes liver metastasis and oxaliplatin resistance in colorectal cancer through LUZP1 degradation and MYL9 phosphorylation

**DOI:** 10.1186/s40164-026-00771-7

**Published:** 2026-04-05

**Authors:** Ruijia Zhang, Wenqin Luo, Qikai Zhou, Dongguo Liang, Yuankai Hao, Fan Chen, Yulin Qiu, Yixian Cao, Zezhi Shan, Yu Zhang, Qingguo Li, Sanjun Cai, Dakui Luo, Shaobo Mo, Bin Ma, Xinxiang Li

**Affiliations:** 1 Department of Colorectal Surgery, Fudan University Shanghai Cancer Center, Shanghai, 200032 China; 2 Department of Oncology, Shanghai Medical College, Fudan University, Shanghai, 200032 China; 3 School of Biomedical Engineering, Med-X Research Institute, Shanghai Jiao Tong University, Shanghai, China; 4 Shanghai Institute of Hematology, National Research Center for Translational Medicine, State Key Laboratory of Medical Genomics, Ruijin Hospital , Shanghai Jiao Tong University School of Medicine, Shanghai, China; 5 Shanghai Key Laboratory for Cancer System Regulation and Clinical Translation, Shanghai Jiading District Central Hospital, Shanghai, China

## Abstract

Colorectal cancer liver metastasis (CRLM) and chemotherapy resistance remain major clinical challenges, with the underlying molecular mechanisms yet to be fully elucidated. In this study, based on analyses of five initial clinical cohorts from FUSCC, the E3 ubiquitin ligase COP1 was identified as a critical driver of CRLM and resistance to oxaliplatin-based chemotherapy. Using an organoid biobank derived from paired primary colorectal tumors and liver metastases, integrated multi-omics analyses (WES, bulk RNA-seq, scRNA-seq) of patient-derived organoids (PDOs) from CRLM revealed significantly elevated COP1 expression in liver metastases compared to primary tumors. High COP1 levels were associated with poor prognosis, increased liver metastatic burden, and resistance to oxaliplatin-based chemotherapy. In vitro and in vivo functional experiments demonstrated that COP1 facilitates CRLM progression by ubiquitinating and degrading LUZP1, thereby releasing DAPK3 from LUZP1-mediated suppression. This process leads to enhanced MYL9 phosphorylation and activation of epithelial-mesenchymal transition (EMT) as well as the JAK2-STAT3-CCND2 signaling axis—pathways crucial for liver metastasis and resistance to oxaliplatin-based chemotherapy. These findings establish the COP1-LUZP1-MYL9 axis as a therapeutic target for CRLM and oxaliplatin-based chemoresistance. Clinically, COP1 expression profiling in PDOs from postoperative specimens enables a precision strategy for managing oxaliplatin-based chemoresistance, especially in the context of FOLFOX.

**Supplementary Information:**

The online version contains supplementary material available at 10.1186/s40164-026-00771-7.

## Introduction

Colorectal cancer (CRC) represents a formidable global health burden, ranking among the top three most frequently diagnosed malignancies and standing as the second leading cause of cancer-related mortality worldwide [1, 2]. While early-stage CRC patients benefit significantly from advances in multidisciplinary care—with over 90% achieving five-year survival—the prognosis for individuals with advanced disease remains bleak, particularly following the onset of distant metastasis occurs, where the five-year survival rate drops below 15% [3, 4]. Alarmingly, approximately 20% of patients present with metastases at initial diagnosis, underscoring the urgent need to dissect the molecular mechanisms that govern CRC dissemination [5, 6].

Among metastatic sites, liver is most commonly affected, with synchronous liver metastases (LM) detected in roughly one-fifth of newly diagnosed CRC cases [7]. Moreover, over half of patients who relapse postoperatively develop liver metastases. Surgical resection combined with systemic chemotherapy remains the cornerstone of treatment for colorectal cancer liver metastasis (CRLM) [8, 9]. However, the optimal therapeutic regimen is still under active investigation due to biological complexity and heterogeneity of CRLM [10]. Notably, the clinical benefit of adjuvant therapies in this context remains contentious.

Currently, 5-fluorouracil (5-FU) and oxaliplatin are frontline chemotherapeutic agents for CRLM [10, 11]. Clinical studies have demonstrated that patients receiving FOLFOX—a combination of 5-FU/leucovorin with oxaliplatin—exhibit superior overall survival (OS) and progression-free survival (PFS) compared to those treated with 5-FU/leucovorin alone [12]. Nevertheless, a substantial subset of CRLM patients fails to respond effectively, reflecting a critical barrier posed by intrinsic or acquired chemoresistance [13]. Oxaliplatin and 5-FU play central roles in CRC chemotherapy; however, resistance to oxaliplatin or 5-FU frequently emerges during treatment, contributing to tumor progression, recurrence, and poor clinical outcomes [14, 15]. This highlights an urgent need to delineate the molecular basis of oxaliplatin or 5-FU resistance and develop strategies to restore chemotherapeutic sensitivity.

Constitutive photomorphogenic 1 (COP1, also known as RFWD2) is a RING finger-type E3 ubiquitin ligase that regulates protein homeostasis through substrate-specific ubiquitination and degradation. Structurally, COP1 harbors an N-terminal RING domain essential for E2 enzyme interaction, a central coiled-coil motif facilitating dimerization, and C-terminal WD40 repeats responsible for substrate recognition. Functionally, COP1 targets several oncogenic and tumor-suppressive proteins—either directly or via recruitment into multiprotein E3 ligase complexes such as CUL4A-DDB1-DET1-RBX1—to regulate key processes including cell proliferation, apoptosis, and DNA repair [16, 17].

Our previous research has elucidated the mechanism by which the CUL4B-DDB1-COP1 complex promotes CRC progression through the ubiquitin-mediated degradation of UTX. As a key E3 ubiquitin ligase within the CUL4B-DDB1 complex, COP1 (RFWD2) directly interacts with UTX and facilitates its ubiquitination and degradation. Knockout of COP1 significantly enhanced UTX stability in CRC cells, leading to reduced CRC cell proliferation, confirming its oncogenic role through UTX degradation [18]. Additionally, Xiao et al. identified COP1 as one of five candidate genes associated with poor prognosis through differential gene expression analysis in circulating tumor cells (CTCs) from patients with metastatic cancers, suggesting a potential role for COP1 in tumor metastasis [19]. However, the role of COP1 in regulating tumor cell invasion and metastasis—particularly in the context of CRLM—remains unexplored, highlighting the need for in-depth mechanistic investigation.

Our research group previously established a paired patient-derived organoid (PDO) library from primary colorectal tumors and matched liver metastases, along with corresponding clinical drug sensitivity assays, including monotherapies with oxaliplatin, 5-FU, or irinotecan, as well as combination therapy (FOLFOX) [20]. Utilizing this well-established organoid platform, we aimed to clarify the regulatory role of COP1 in liver metastasis and further integrate clinical drug response data to determine whether COP1 is implicated in chemoresistance in colorectal cancer.

Here, we elucidated the mechanistic role of COP1 in CRLM and chemoresistance through comprehensive in vitro and in vivo experiments combined with clinical analysis. Importantly, our findings established a preclinical foundation that may pave the way for future exploration of COP1-targeting inhibitors as a potential therapeutic strategy in CRC management.

##  Methods

### Organoid culture

All procedures involving human specimens were performed following ethical approval granted by the Ethics Committee of Fudan University Shanghai Cancer Center (FUSCC) (approval ID: 050432-4‐2108*). Resected samples of primary colorectal cancer (CRC) and matched liver metastatic lesions were acquired from patients who underwent combined intestinal and hepatic surgery at the Department of Colorectal Surgery. Postoperative clinical information—including imaging data from computed tomography (CT) and magnetic resonance imaging (MRI), as well as follow-up records—was retrospectively retrieved from the institutional medical database. All research activities complied with internationally accepted ethical standards, including the Declaration of Helsinki, and written informed consent was obtained from each participant prior to inclusion. After surgical excision, representative tissue blocks—comprising non-malignant colonic mucosa, primary tumor, and corresponding liver metastasis, each exceeding 1 cm in diameter—were rapidly processed. Each specimen was promptly sectioned into three portions. One was immersed in pre-cooled phosphate-buffered saline (PBS) supplemented with antibiotics (Solarbio, P1400) and transported on ice for organoid derivation. The second was snap-frozen in liquid nitrogen for molecular analyses, while the third was fixed in 4% paraformaldehyde (Sigma Aldrich, P6148) for histological evaluation.

Organoids derived from both primary CRC and metastatic liver tissues were imaged at appropriate time points. For routine passaging, the Matrigel domes containing organoids were transferred into 15 mL conical tubes with ice-cold PBS and pelleted by centrifugation at 200×g. After removal of the supernatant, the pellet was resuspended and mechanically dissociated by pipetting 30–60 times using a 1mL pipette. The resulting single-cell suspension was embedded in fresh Matrigel at a 1:2 ratio and plated for continued culture, with medium refreshed every 3 days. For long-term storage, organoids were cryopreserved in serum-free freezing medium (CELLBANKER™ 2, ZENOAQ, 170905). During revival, 10µM Y-27,632 was added to the CRLM culture medium to enhance organoid viability. The CRLM-specific culture medium was based on Advanced DMEM/F12 and supplemented with a comprehensive set of growth factors and signaling modulators, including R-spondin 1, Noggin, EGF, HEPES, Glutamax, Normocin, Gentamicin/Amphotericin B, N2, B27, N-acetyl-L-cysteine, Nicotinamide, ALK4/5/7 inhibitor, p38 MAPK inhibitor, Gastrin, and Prostaglandin E2. The clinicopathological characteristics of the five paired CRLM PDOs included in this study are summarized in Supplementary Table S1. These patients were collectively designated as FUSCC Cohort I. All five cases represented synchronous colorectal liver metastases.

### RNA sequencing and whole exome sequencing

Well-established organoid cultures were subjected to total RNA extraction using TRIzol reagent, following the manufacturer’s protocol, for subsequent transcriptomic profiling. RNA integrity and quality control steps included electrophoretic assessment on 1% agarose gels to detect degradation and contamination, spectrophotometric measurement of purity via NanoPhotometer (IMPLEN, CA, USA), and integrity evaluation using the RNA Nano 6000 Assay Kit on the Agilent Bioanalyzer 2100 system (Agilent Technologies, CA, USA). For RNA-seq, expression data from cell lines were normalized using FPKM, while data from PDOs of FUSCC were normalized as TPM. Gene expression levels quantified as TPM or FPKM were log2-transformed using log2(TPM/FPKM + 1) before subsequent analyses. Expression data of PDOs were protected due to patient privacy considerations but were available from the corresponding author upon reasonable request. FPKM expression matrices for HCT116 cells with COP1 overexpression and the oxaliplatin-resistant cell line, are available in Supplementary Table S2. For multi-sample integration, batch effects in bulk RNA-seq were corrected using the ComBat algorithm.

For genomic DNA extraction, organoids grown under optimal conditions in 24 well plates were collected and cryopreserved prior to WES library preparation. Matched tumor DNA was isolated from corresponding frozen tumor tissues stored in liquid nitrogen, while germline DNA was obtained from cryopreserved non-neoplastic colonic tissue samples. Transformation of Whole-Exome Sequencing (WES) data into Mutation Annotation Format (MAF) entails subjecting raw sequencing outputs (FASTQ or BAM files) to standardized somatic variant-calling workflows, such as GATK/MuTect2-based pipelines, to generate variant call files (VCFs). These variants are subsequently functionally annotated and reformatted into the tab-delimited MAF structure, which consolidates key mutational attributes—including affected genes, mutation classes, allele frequencies, and predicted functional impacts—thereby facilitating efficient mutation filtering and downstream analyses in cancer genomics.

### Preparation of bulk RNA public datasets

Bulk RNA sequencing datasets for colorectal cancer, together with corresponding clinical annotations, were retrieved from publicly available repositories, including the Gene Expression Omnibus (GEO) and The Cancer Genome Atlas (TCGA). Specifically, gene expression profiles from 913 patients across three GEO cohorts (GSE39582 [21], GSE14333 [22], and GSE37892 [23]) were included for integrative analysis. To mitigate technical variability arising from different studies, batch effects were corrected using the ComBat algorithm, after which the three datasets were merged to construct a unified meta-GEO cohort for downstream analyses.

### Single cell RNA sequencing and analyses

PDOs were dissociated into single-cell suspensions for downstream sequencing. Single-cell capture and library construction were performed using the Chromium Single Cell 3’ platform (10x Genomics), following the standard operating procedures provided by the manufacturer. Each sample was loaded onto the Chromium Controller along with gel beads and reagents to generate gel bead-in-emulsion (GEM) droplets, enabling individual cell barcoding. Approximately 5,000 cells per sample were targeted for capture. Subsequent cDNA amplification and library preparation were evaluated using the High Sensitivity DNA Kit on the Agilent BioAnalyzer (Agilent Technologies) to ensure quality and fragment distribution. Libraries were pooled and sequenced on an Illumina NovaSeq 6000 system, achieving a depth of roughly 400 million reads per sample.

Raw BCL files were demultiplexed and converted to FASTQ format using Illumina’s bcl2fastq software (v2.19.1). Reads were aligned to the GRCh38 human genome reference, and count matrices were generated using the Cell Ranger software suite (v4.0.0, 10x Genomics), which performs barcode assignment and gene expression quantification. The resulting gene-by-cell matrices were processed in Seurat for downstream analysis.

### Quality control and analysis of single-cell RNA sequencing data

Downstream analyses were subsequently performed using the Seurat package. Quality control filtering was applied based on the number of detected genes per cell (> 100) and the proportion of mitochondrial genes (< 50%). Single-cell gene expression data were normalized using Seurat’s normalization workflow, in which expression values for each cell were scaled according to total transcript counts, adjusted to a fixed scaling factor (default 10,000), and subsequently log-transformed. Genes exhibiting high expression variability across cells were selected and carried forward for downstream dimensionality reduction by principal component analysis.

To integrate single-cell datasets from multiple samples and mitigate batch effects, we applied Harmony integration, a method that effectively minimizes technical variation while retaining underlying biological signals across batches. The Harmony-corrected embeddings were incorporated into the Seurat object, and subsequent dimensionality reduction and re-clustering were performed using these Harmony embeddings instead of the original principal components by specifying reduction = “harmony” with standard Seurat analysis parameters. Pathway activity scoring for scRNA-seq data was performed using the AddModuleScore function implemented in Seurat, with gene sets derived from the Gene Ontology (GO) Biological Process (BP) database.

### Immunohistochemistry (IHC)

In this study, patients involved in IHC analysis were categorized into five cohorts: FUSCC Cohort I, FUSCC Cohort II consisting of 20 pairs of matched CRLM tissue sections, FUSCC Cohort III comprising a tissue microarray (TMA) of 99 liver metastatic lesions from CRLM patients without neoadjuvant therapy, FUSCC Cohort IV including a TMA of 168 primary CRC tumors (with prognostic information available for 166 cases), and FUSCC Cohort V consisting of a TMA of 101 liver metastases from CRLM patients who received preoperative chemotherapy. The corresponding primary tumor specimens for the two liver metastasis TMA cohorts were not collected or included for TMA construction.

Tumor specimens were sourced from FUSCC. Ethical approval was granted by the Institutional Ethics Committee of FUSCC, and all participants provided written informed consent in accordance with institutional guidelines. Immunohistochemical staining was conducted following established procedures. In brief, formalin-fixed, paraffin-embedded tissues were sectioned at 4μm thickness and mounted on polysine-coated glass slides. The slides were baked at 58°C overnight, deparaffinized with xylene, and rehydrated through a graded ethanol series. Endogenous peroxidase activity was blocked using 0.3% hydrogen peroxide for 15 min. Antigen retrieval was achieved by pressure cooking the sections in citrate buffer (pH 6.0) for 20 min, followed by cooling to room temperature. To reduce nonspecific binding, sections were incubated in 5% normal goat serum for 2 h. Primary antibody incubation was performed at 37 °C for 1.5 h using antibodies against COP1 (1:100, Bethyl, A300-894), LUZP1 (1:100, Protentech, 17483-1-AP), MKI67 (1:200, Cell Signaling Technology, #9129) and CCND2 (1:200, Abcam, EPR19659).

We implemented an objective, software-based quantification method to evaluate IHC score. We employed the Saiviewer immunohistochemistry analysis platform to automatically calculate an immunoreactivity score (IRS). The IRS is determined by multiplying a score for the percentage of positively stained area (0: <5%; 1: 5–25%; 2: 26–50%; 3: 51–75%; 4: >75%) by a score for staining intensity (0: negative; 1: weak; 2: moderate; 3: strong). All scores were generated algorithmically by the software. An IRS > 6 was established as the optimal cutoff to define the “COP1-high” group.

### Cell culture

The human CRC cell lines HCT15, DLD1, HCT116, SW480, LoVo, and RKO; the murine CRC cell line MC38; and the human embryonic kidney cell line 293T were obtained from the National Cancer Institute (Bethesda, MD, USA). All CRC and 293T cells were cultured in Dulbecco’s Modified Eagle Medium (DMEM; SH30243.01, HyClone, Logan, UT, USA) supplemented with 10% fetal bovine serum (FBS; 10099141, Thermo Fisher Scientific, Waltham, MA, USA). All cell lines used in this study were authenticated by short tandem repeat (STR) profiling to confirm their identity.

### Construction of stable cell lines

Short hairpin RNAs (shRNAs) targeting COP1, LUZP1, DAPK3, DET1, DDB1, CUL4B and CCND2 were obtained from Genechem (Shanghai, China). The lentiviral overexpression vectors for COP1, LUZP1, and CCND2 were constructed and utilized. The GV369 vector, carrying a GFP reporter, was driven by the ubiquitin promoter and used for stable overexpression. The GV341 vector, harboring a FLAG tag and also driven by the ubiquitin promoter, was primarily used for co-immunoprecipitation (co-IP) assays. In addition, the pCDH-CMV-HA-Puro vector, which carries an HA tag, was driven by the CMV promoter and employed for functional and mechanistic studies. All constructs were verified by Sanger sequencing.

To produce lentiviral particles, the recombinant plasmids were co-transfected with packaging plasmids (psPAX2 and pMD2.G) into 293T cells using Lipofectamine 3000 (L3000008, Thermo Fisher Scientific) according to the manufacturer’s instructions. After 48h of incubation, viral supernatants were collected and subsequently used to infect target cell lines. Lentiviral particles were applied to: (a) RKO and SW480 cells for stable COP1 overexpression and empty vector; (b) HCT15 and DLD1 cells for stable COP1 knockdown and negative control; (c) RKO and SW480 cells for stable LUZP1 knockdown and negative control; (d) HCT15 and DLD1 cells for stable LUZP1 overexpression and empty vector; (e) RKO cells for stable CCND2 overexpression and empty vector; (f) DLD1 cells for stable CCND2 knockdown and negative control. Successfully transduced cells were selected using puromycin to establish stable cell lines.

### Wound healing assay

A total of 1 × 10⁵ cells were seeded per well in six-well plates, with three replicates prepared for each condition. After the cells formed a uniform monolayer across the well surface, linear wounds were introduced by dragging sterile 10µL pipette tips across the cell layer. Non-adherent cells were removed by gentle washing, and the cultures were maintained in serum-free medium. At 24h following scratch induction, five randomly chosen areas per well were imaged using bright-field microscopy, and cell migration was assessed by manual counting.

###  Invasion assay

For invasion assay, 5 × 10⁴ cells per well were suspended in serum-free medium and seeded into the upper chambers of 24-well transwell inserts pre-coated with Matrigel, followed by a 48h incubation period. For both migration and invasion analyses, cells that remained in the upper compartment were removed after incubation. The membranes were then fixed in 4% paraformaldehyde at room temperature for 30 min, stained with 0.5% crystal violet for another 30 min, and rinsed thoroughly with PBS. Cells that had traversed to the underside of the membrane were visualized and photographed using an Olympus light microscope. Quantification was performed by counting cells in three randomly selected fields from eight equal regions. Statistical differences between two groups were evaluated using a two-tailed Student’s t-test.

For the 3D invasion assay, collagen-based matrices were freshly prepared by combining rat-tail collagen (Corning, catalog no. 354236) with 10× phosphate-buffered saline (PBS) following the manufacturer’s recommendations. PDOs were then gently mixed into the collagen solution, accounting for approximately one quarter of the final volume, and the mixture was transferred onto culture plates that had been pre-equilibrated at 26 °C for 48h. To ensure uniform distribution of organoids and proper gel formation, the plates were carefully inverted for approximately 8 min. After complete polymerization of the collagen network, gels were incubated at 26 °C for an additional 75 min, followed by the addition of culture medium. Organoids were subsequently maintained under invasion-permissive conditions for 72h before analysis [24].

### Sphere formation assay

To investigate the roles of COP1 and CCND2 in regulating cancer stem-like properties, a tumor sphere formation assay was performed. HCT116-derived stable cell populations were collected, rinsed twice with PBS, and enumerated. A total of 500 cells were seeded per well in 96-well plates containing 200µL of a defined serum-free stem cell medium. This medium consisted of a 3:1 mixture of DMEM and F12 (Thermo Fisher Scientific), supplemented with 0.4% BSA (Sangon), 0.2× B27 (Thermo Fisher Scientific), 10ng/mL recombinant EGF (Sino Biological Inc.), and 10 ng/mL bFGF (PeproTech, Jiangsu, China). After a 3-day incubation period, spheroid formation was documented using microscopy.

###  Immunoprecipitation and western blot analysis

Forty-eight hours following transfection with the designated plasmids, cells were harvested and lysed in a buffer containing 50mM Tris (pH 7.4), 150mM NaCl, 1mM EDTA, 0.25% Triton X-100, and a protease inhibitor cocktail. The lysates were clarified by centrifugation at 12,000 × g for 15 min, and the supernatants were divided into two portions. One portion was incubated with either anti-Flag (Sigma) or anti-COP1 antibodies, while the other was treated with control IgG at 4 °C for 2 h. Subsequently, Protein G-conjugated agarose beads (Roche) were added to each reaction and rotated for an additional hour. The resulting immune complexes were washed four times with lysis buffer, resolved by SDS-PAGE, and analyzed by Western blot or liquid chromatography–tandem mass spectrometry (LC–MS/MS).

We performed quantitative analysis of the Western blot signals using SWE Image Gray Analysis Software. Integrated Density Values (IDVs) for COP1 and LUZP1 were calculated and normalized to the IDV of Actin using the formula IDV (target)/IDV (loading control).

### IP-MS methodology and candidate prioritization

293T cells were transfected with COP1-FLAG or empty vector (Vector-FLAG) plasmids. After 48 h, cells were lysed, and the supernatants were subjected to immunoprecipitation using an anti-FLAG antibody. The captured complexes were analyzed by LC-MS/MS. The experimental design included one replicate per condition (COP1-FLAG *n* = 1, Vector-FLAG *n* = 1) for the initial screening. Only proteins with no detectable signal in the Vector-FLAG control (Intensity = 0) were considered. Within this subset, proteins detected in the COP1-FLAG sample were ranked in descending order based on their intensity values, and the top 50 were selected as high-confidence interactors (Supplementary Table S3).

### In vivo xenograft and liver metastasis models

Animal Model and Group Allocation: All xenograft and liver metastasis experiments utilized female mice to control for sex-based variability. Animals were age- and weight-matched at the start of each experiment and randomly allocated to experimental or control groups to minimize bias. Sample Size and Blinding: Each treatment group contained at least *n* ≥ 3 animals to ensure biological replication. All outcome assessments, including tumor measurement, bioluminescence quantification, and histological analysis, were performed by researchers blinded to group assignments. In accordance with our institutional animal care protocol, mice bearing subcutaneous tumors exceeding a maximum diameter of 20 mm, or showing signs of significant distress, were humanely euthanized and excluded from subsequent tumor growth analyses. This pre-defined criterion was applied to all cohorts.

To evaluate tumorigenic potential in vivo, CRC cells (1 × 10⁶) were resuspended in 100µL of a 1:1 mixture of serum-free medium and Matrigel, and subcutaneously implanted into the right flanks of 6-week-old male BALB/c nude mice (SLAC, Shanghai, China). Tumor growth was monitored, and volumes were estimated using the formula: volume = 0.5 × length × width². All animals were maintained under SPF conditions in a temperature- and humidity-controlled facility. Animal procedures were conducted in accordance with institutional guidelines and approved by the Animal Ethics Committee of Fudan University.

For drug administration, HS94 was delivered via tail vein injection at a dose of 15mg/kg per mouse, while oxaliplatin was administered intraperitoneally at a concentration of 5 mg/kg per mouse. The oxaliplatin dose (5mg/kg, i.p.) was selected based on its established efficacy and tolerability in prior murine studies [25]. The HS94 dose (15mg/kg, i.v.) was chosen as a representative and effective dose within the 10–20mg/kg range reported for DAPK3 inhibitors in previous mouse model studies [26]. Oxaliplatin was prepared in phosphate-buffered saline (PBS). HS94 was formulated in a vehicle consisting of 10% DMSO, 40% PEG300, 5% Tween-80, and 45% saline.

For liver metastasis modeling, luciferase-transfected CRC cells were introduced into the spleens of either NOD scid gamma (NSG) or C57BL/6 mice. Mice were sacrificed 3 weeks post-injection using an overdose of pentobarbital, and liver tissues were harvested for subsequent analysis.

### Drug assays in PDOs and dose-response curve fitting methodology

For drug preparation, oxaliplatin (MCE: HY-17371) and 5-FU (MCE: HY-90006) was dissolved in sterile water. SN-38 (the active metabolite of irinotecan; MCE: HY-13704) and HS94 (MCE: HY-120040) was dissolved in DMSO.

To ensure comparability and interpretability of drug-sensitivity data from PDOs in FUSCC Cohort I, all drug-response assays were performed under the same experimental batch conditions. Organoids were treated with single agents including oxaliplatin, 5-fluorouracil (5-FU), and SN-38 (the active metabolite of irinotecan), and the corresponding IC₅₀ values were calculated and summarized in Supplementary Table S4. All dose–response curves were fitted using nonlinear regression (log[inhibitor] vs. response, variable slope) in GraphPad Prism.

Where appropriate, constrained curve-fitting models were applied. Specifically, for datasets in which the tested concentration range sufficiently spanned the dynamic response range (e.g., reaching clear plateaus at both high and low concentrations), curves were constrained to a top plateau of 100% viability (vehicle control) and a bottom plateau of 0% viability, thereby yielding more robust and biologically interpretable IC₅₀ estimates. For datasets in which the concentration range did not achieve complete inhibition, an unconstrained variable-slope model was used to interpolate IC₅₀ values based on the observed data points.

###  FOLFOX treatment in PDOs

A series of 5-FU or oxaliplatin monotherapy treatments were prepared using a concentration gradient of 50, 20, 10, 5, 1, 0.5, and 0µM. For combination therapy with FOLFOX (5‐FU: leucovorin: oxaliplatin = 25:5:1), the 5‐FU component followed the same concentration range, with corresponding proportional adjustments of leucovorin and oxaliplatin. All concentrations were tested in triplicate to ensure reproducibility.

### Statistical analysis

All statistical analyses were performed using GraphPad Prism (version 10.0) and R software (version 4.3.3). Specific R packages are cited where applicable (e.g., survival for Cox models, clusterProfiler for enrichment analyses). Comparisons between two groups were made using two-tailed Student’s t-tests, applied when data met assumptions of normality and homogeneity of variance (assessed via Shapiro-Wilk and F tests, respectively). Non-parametric alternatives (e.g., Mann-Whitney U test) were used when assumptions were violated. Associations were assessed using Pearson correlation analysis. Survival differences were compared with the log-rank test. Multivariable Cox proportional hazards models were employed for adjusted analyses. For high-dimensional data (e.g., RNA-seq differential expression, pathway enrichment), false discovery rate (FDR) correction was applied using the Benjamini-Hochberg method. Adjusted *P* values (FDR) are reported for these analyses.

## Results

### Elevated COP1 expression in colorectal cancer liver metastases predicts poor prognosis

**Fig. 1:**
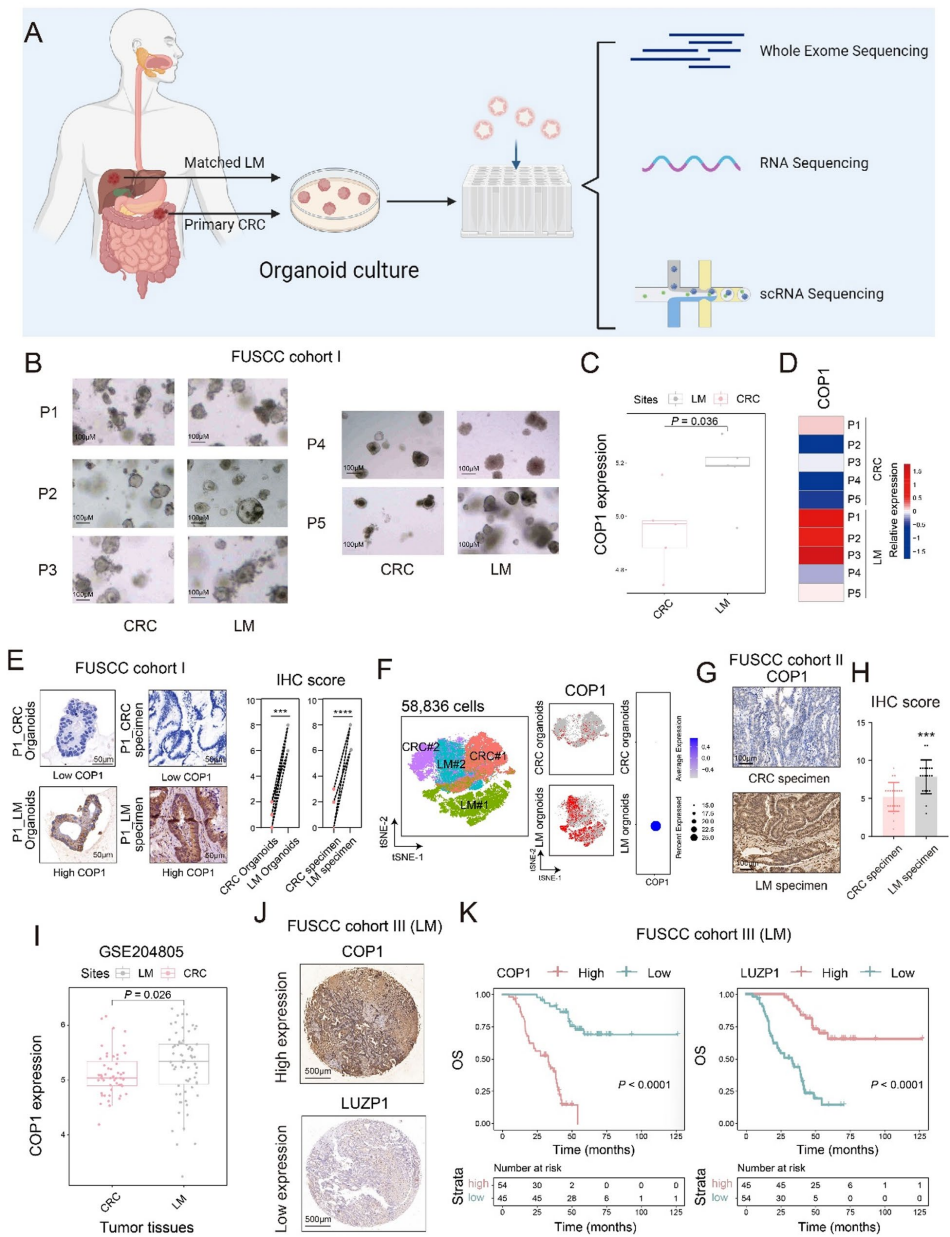
Elevated COP1 expression in colorectal cancer liver metastases predicts poor prognosis. **A**. Construction of a patient-derived organoid (PDO) library from CRLM specimens, followed by multi-omics analyses—including WES, RNA sequencing and scRNA sequencing—was performed. **B**. Morphological presentation of paired CRLM organoids under bright-field microscopy; scale bar = 100μm. **C**. Box plot showing the differential gene expression of COP1 between liver metastases and matched primary tumors in paired CRLM PDOs (each group *n* = 5); t-test. **D**. Heatmap illustrating COP1 expression levels in liver metastases and matched primary tumors of paired CRLM PDOs (each group *n* = 5); t-test. **E** IHC showing COP1 protein expression levels in liver metastases and matched primary tumors of CRLM PDOs and their corresponding tumor specimen (each group *n* = 5); scale bar = 50μm. **F** t-SNE visualization of single cells from CRC and matched LM samples, comprising a total of 58,836 cells. Cells are colored by sample origin (two pairs of CRLM), as indicated. Feature plots showing COP1 expression in CRC and LM cells projected onto the t-SNE space, and dot plot summarizing the average expression level and the proportion of COP1-expressing cells in each group. **G** Representative IHC images showing COP1 expression in primary CRC and paired LM tissues from the FUSCC Cohort II. Scale bar, 100μm. **H** Quantification of COP1 IHC scores in primary lesions and liver metastatic tumors. Data are presented as mean ± SD, with individual data points shown; t-test. **I** Analysis of COP1 expression in primary CRC tissues and matched LM from the public dataset GSE204805. Box plots show COP1 expression levels in CRC (*n* = 51) and LM samples (*n* = 68); t-test. **J** Representative immunohistochemical staining showing high and low expression of COP1 and LUZP1 in liver metastasis specimens from the FUSCC Cohort III. Scale bar, 500 μm. **K** Kaplan-Meier overall survival (OS) analysis of patients with CRLM (FUSCC Cohort III), stratified by COP1 or LUZP1 IHC scores; log-rank test. *** *P* < 0.001

By establishing an organoid biobank derived from paired primary colorectal tumors (CRC) and matched liver metastases (LM), we conducted integrated multi-omics profiling including WES, bulk RNA-seq, and scRNA-seq on these organoids, thereby generating a robust dataset to underpin mechanistic investigations in this study (Fig. 1A). From this biobank, we successfully derived and maintained five paired PDO cultures from patients with colorectal cancer liver metastases (CRLM), which were designated as FUSCC Cohort I (Fig. 1B). The associated clinical information is provided in Supplementary Table S1. WES analysis of three paired patient tissues and their corresponding PDOs (P1, P2, and P5) (Figure S1A) revealed a high concordance of mutations between organoids and patient tissues. This concordance indicated that PDOs could effectively recapitulate expression differences between primary and liver metastatic tumors.

Firstly, transcriptomic analysis of CRLM PDOs revealed that COP1 expression was significantly higher in LM organoids than in their matched CRC organoids (*P* = 0.036, by t-test, Fig. 1C). A heatmap further illustrated the distribution of COP1 expression across the ten PDO samples (Fig. 1D). To validate these transcriptional findings at the protein level, we performed COP1 IHC staining in five paired PDOs together with their corresponding source tumor specimen sections (Fig. 1E and Figure S1B). Consistent with the mRNA results, COP1 protein expression was higher in LM PDOs than in CRC PDOs, and the corresponding LM specimen likewise exhibited higher COP1 expression than the matched CRC specimen. We next expanded the dataset by incorporating two pairs of matched CRLM-derived PDOs for scRNA-seq analysis (Fig. 1F and Figure S2C). Following unsupervised clustering, a total of 18 distinct cell clusters were identified (Figure S2C). To confirm the epithelial tumor identity of the organoid cells, expression of the epithelial marker EPCAM was examined across all cells (Figure S2D). Consistent with the bulk and protein-level analyses, COP1 expression was elevated in LM organoids compared with CRC organoids (Fig. 1F and Figure S2E). To extend these observations to clinical samples, we analyzed an independent cohort of 20 matched CRLM patient tissues (designated as FUSCC Cohort II; Fig. 1G). IHC staining demonstrated significantly higher COP1 expression in liver metastatic lesions than in the corresponding primary tumors (*P* < 0.001, by t-test; Fig. 1G and H). To move beyond our institutional cohorts, we performed an independent validation using the public dataset GSE204805 [27], which includes matched primary and liver metastatic (LM) tissues, as well as patient-derived xenograft (PDX) models. Our analysis confirmed that COP1 mRNA expression is significantly higher in LM tissues (*n* = 68) compared to primary tumors (*n* = 51) (*P* = 0.026, by t-test; Fig. 1I). Critically, this elevation was recapitulated in a much larger set of PDX models, with LM-derived PDXs (*n* = 664) showing markedly higher COP1 expression than primary tumor-derived PDXs (*n* = 159) (Figure S1C). This robust external validation strongly supports the consistent upregulation of COP1 in colorectal cancer liver metastases. We further validated the prognostic relevance of COP1 expression using a meta-GEO cohort (Figure S1D-S1E). High COP1 expression was associated with significantly poorer overall survival and was preferentially enriched in KRAS-mutant while depleted in dMMR/MSI-high tumors, supporting its link to aggressive tumor biology in colorectal cancer.

Next, we analyzed COP1 expression in a LM tissue microarray (TMA), designated as FUSCC Cohort III, via IHC staining (Fig. 1J). Baseline clinical characteristics (Table 1) showed no significant differences in gender, age, primary tumor location, pathological type, or molecular features (MSI/MSS status, KRAS mutation, and BRAF^V600E^ mutation) between the two groups, indicating that these variables did not influence the grouping. However, patients in the high COP1 expression group exhibited a higher prevalence of N2 lymph node staging, suggesting that COP1 overexpression was associated with an increased risk of lymphatic metastasis (*P* = 0.0362, by chi-square test; Table 1).

**Table 1:** Clinical feature analysis based on COP1 and LUZP1 expression groups in CRLM-TMA (FUSCC III)

Characteristics	*N*	COP1			LUZP1		
		High expression group (*N* = 54), %	Low expression group (*N* = 45), %	*P* value	High expression group (*N* = 45), %	Low expression group (*N* = 54), %	*P* value
Gender, n (%)				0.8396			0.7234
Male	58	31(57.4%)	27(60.0%)		25 (55.6%)	33 (61.1%)	
Female	41	23(42.6%)	18(40.0%)		20 (44.4%)	21 (38.9%)	
Age, n (%)				0.4247			0.8110
≤ 60	46	23(42.6%)	23(51.1%)		22 (48.9%)	24 (44.4%)	
> 60	53	31(57.4%)	22(48.9%)		23 (51.1%)	30 (55.6%)	
Primary site, n (%)				0.6437			0.2224
Colon	75	42(77.8%)	33(73.3%)		31 (68.9%)	44 (81.5%)	
Rectum	24	12(22.2%)	12(26.7%)		14 (31.1%)	10 (18.5%)	
Histological type, n (%)				0.5383			0.5383
Adenocarcinoma	87	46(85.2%)	41(91.1%)		41 (91.1%)	46 (85.2%)	
Mucinous/SRCC	12	8(14.8%)	4(8.9%)		4 (8.9%)	8 (14.8%)	
pT stage, n (%)				0.2119			0.6193
T1/T2	8	2(3.7%)	6(13.3%)		3 (6.7%)	5 (9.3%)	
T3	64	37(68.5%)	27(60.0%)		30 (66.7%)	34 (63.0%)	
T4	27	15(27.8%)	12(26.7%)		12 (26.6%)	15 (27.7%)	
pN stage, n (%)				0.0362			0.2340
N0	27	13(24.1%)	14(31.1%)		13 (28.9%)	14 (25.9%)	
N1	44	20(37.0%)	24(53.3%)		23 (51.1%)	21 (38.9%)	
N2	28	21(38.9%)	7(15.6%)		9 (20.0%)	19 (35.2%)	
MSI/MSS status				/			/
MSS	96	52(96.3%)	44(97.8%)		44(97.8%)	52(96.3%)	
MSI	0	0(0.0%)	0(0.0%)		0(0.0%)	0(0.0%)	
Not tested	3	2(3.7%)	1(2.2%)		1(2.2%)	2(3.7%)	
KRAS mutation				0.4405			0.0150
Yes	36	19 (35.2%)	17 (46.7%)		21 (46.7%)	15 (27.8%)	
No	42	21 (39.9%)	21 (37.8%)		20 (44.4%)	22 (40.7%)	
Not tested	21	14 (25.9%)	7 (15.5%)		4 (8.9%)	17 (31.5%)	
BRAF^V600E^ mutation				0.3894			0.0303
Yes	1	1(1.9%)	0(0.0%)		0(0.0%)	1(1.8%)	
No	76	39(72.2%)	37(82.2%)		40(88.9%)	36(31.5%)	
Not tested	22	14(25.9%)	8(17.8%)		5(11.1%)	17(66.7%)	

To rigorously control for potential confounding factors, we constructed a baseline Cox proportional hazards (PH) model incorporating 12 core clinicopathological variables, including T stage, N stage, size and number of liver metastases, primary tumor site, gender, age, serum CEA level, histological subtype, MSS/MSI status, KRAS mutation status, and BRAF^V600E^ mutation status, in order to comprehensively adjust for baseline confounders. COP1 expression was then added to this baseline model to generate the full multivariable Cox regression model. In analyses using overall survival (OS) as the endpoint, testing of the proportional hazards assumption indicated that histological subtype violated the proportional hazards assumption. Therefore, a stratified multivariable Cox regression model was applied, with histological subtype included as a stratification factor. The results demonstrated that elevated COP1 expression in liver metastases was significantly associated with poorer OS (Table 2, *P* < 0.001, by stratified multivariable Cox regression). In analyses using disease-free survival (DFS) as the endpoint, proportional hazards assumption testing revealed violations for gender, primary tumor site and N stage. Accordingly, stratified multivariable Cox regression models were employed to account for these violations. Consistently, high COP1 expression in liver metastases remained significantly correlated with poorer DFS (Table 2, *P* < 0.001, by stratified multivariable Cox regression). Finally, Kaplan-Meier survival analysis was performed to evaluate the impact of COP1 expression on OS (Fig. 1K) and DFS (Figure S1F). The results indicated that CRLM patients with higher COP1 expression had significantly worse survival outcomes (all P values were < 0.01, by log-rank test), which was consistent with our multivariate Cox regression findings.

**Table 2:** Multivariate Cox regression analysis of COP1 expression and OS/DFS in CRLM-TMA without chemotherapy (FUSCC III)

Characteristics	Overall survival		Disease free survival	
	HR (95%CI)	*P*	HR (95%CI)	*P*
Gender	0.89 (0.48, 1.65)	0.704	/	/
Age	1.31 (0.68, 2.49)	0.427	1.05 (0.57, 1.9)	0.868
Primary site	0.87 (0.38, 1.98)	0.735	/	/
Histological type, n (%)	/	/	2.99 (1.21, 7.40)	0.018
pT stage, n (%)	0.86 (0.52, 1.42)	0.555	0.71 (0.43, 1.18)	0.185
pN stage, n (%)	1.26 (0.77, 2.06)	0.361	/	/
LM size, n (%)	1.02 (0.89,1.17)	0.771	0.98 (0.88, 1.09)	0.733
LM nodule, n (%)	1.12 (1.01, 1.24)	0.025	1.03 (0.34, 1.14)	0.560
CEA, n (%)	1.00 (1.00, 1.00)	0.527	1.00 (1.00, 1.00)	0.410
KRAS mutation, n (%)	0.75 (0.39, 1.46)	0.407	0.91 (0.52, 1.58)	0.740
BRAF^V600E^ mutation, n (%)	0.96 (0.31, 2.94)	0.939	1.00 (0.41, 2.43)	0.990
MSI/MSS status, n (%)	0.27 (0.11, 0.67)	0.005	0.56 (0.23, 1.35)	0.199
COP1 expression	2.00 (1.62, 2.46)	< 0.001	1.37 (1.18, 1.59)	< 0.001

### Overexpression of COP1 promotes colorectal cancer liver metastasis

**Fig. 2:**
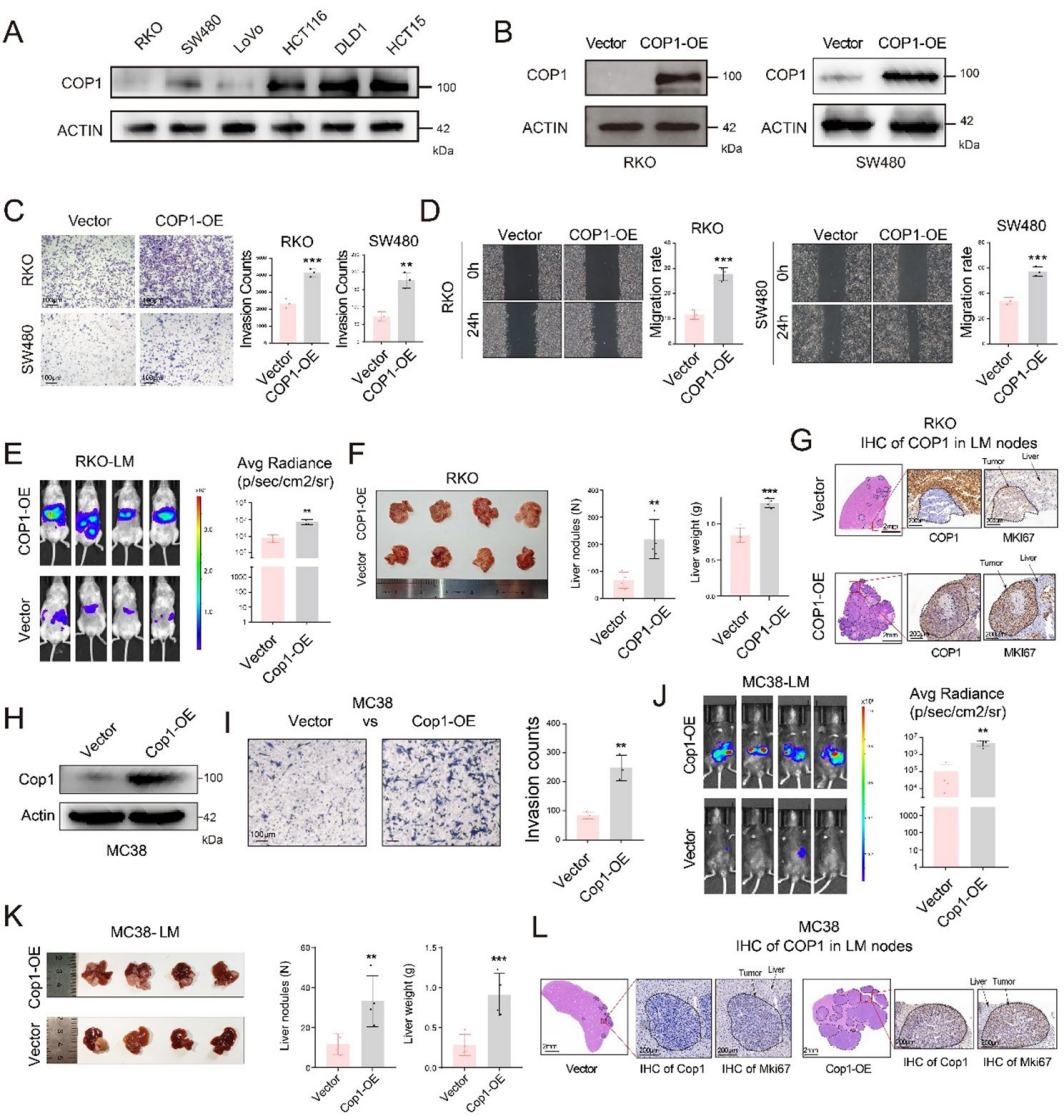
In vitro and in vivo experiments demonstrate that COP1 promoted colorectal cancer liver metastasis. **A** Western blot showing COP1 protein expression levels across six different human CRC cell lines (RKO, SW480, LoVo, HCT116, DLD1 and HCT15). **B** Western blot analysis was performed to assess protein expression level following COP1 overexpression in RKO and SW480 cells. **C** Invasion assays were conducted to compare the invasive capacity of COP1-overexpressing RKO and SW480 cells with their respective vector control cells (each group *n* = 3); scale bar = 100μm. **D** Wound healing assays were performed to evaluate the migratory ability of COP1-overexpressing RKO and SW480 cells compared to their respective vector control cells (each group *n* = 3). **E** A LM mouse model was established using COP1-OE RKO-luciferase and vector control RKO-luciferase in NSG mice. Quantitative analysis of metastatic burden was performed using fluorescence imaging (each group *n* = 4). **F** Representative anatomical images of liver metastases from **E**, accompanied by a bar graph quantifying liver nodules and liver weights (each group *n* = 3). **G** IHC staining of COP1 and MKI67 in liver metastases nodes derived from RKO-Luc cells with vector control or COP1-OE. Representative images of metastatic tumor regions are shown. Scale bars, 2 mm (whole section) and 200μm (magnified views). (H) Western blot analysis was performed to assess protein expression level following Cop1 overexpression in MC38 cells. **I** Invasion assays were conducted to compare the invasive capacity of Cop1-overexpressing MC38 with their respective vector control cells (each group *n* = 3). **J** A LM mouse model was established using Cop1-OE MC38-Luc and vector control MC38-Luc in C57BL/6 mice. Quantitative analysis of metastatic burden was performed using fluorescence imaging (each group *n* = 4). **K** Representative anatomical images of liver metastases from I, accompanied by a bar graph quantifying liver nodules and liver weights. **L** IHC staining of Cop1 and Mki67 in liver metastases nodes derived from MC38 cells with vector control or Cop1-OE. Representative images of metastatic tumor regions were shown. Scale bars, 2mm (whole section) and 200μm (magnified views). Data are presented as mean ± SD, with individual data points shown; t-test. ** *P* < 0.01, *** *P* < 0.001

Functional enrichment analysis of RNA-seq data from organoids revealed that samples with high COP1 expression were significantly enriched for gene signatures related to cell migration, including wound healing and extracellular matrix organization (Figure S2A). Further analysis of scRNA-seq data from organoids confirmed that LM-derived organoids with high COP1 expression also exhibited elevated gene scores associated with wound healing and extracellular matrix organization (Figure S2F and S2G). To validate these findings, RKO and SW480 cells with low COP1 expression were selected for overexpression experiments (Fig. 2A and B). Transwell assays demonstrated that COP1 overexpression (COP1-OE) significantly enhanced the invasive capacity of CRC cells (Fig. 2C, *P* < 0.01, by t-test). Consistently, wound healing assays showed that COP1-OE significantly promoted cell migration (Fig. 2D, *P* < 0.001, by t-test). Next, we established a liver metastasis model by injecting RKO-luciferase (RKO-luc) cells into the spleens of immunodeficient mice. Bioluminescence imaging revealed that COP1-OE RKO-luc exhibited significantly greater liver metastasis compared to control RKO (Fig. 2E, *P* < 0.01, by t-test). Gross anatomical examination showed that, compared with the Vector group, the COP1-OE group developed a significantly greater number of liver metastatic nodules (Fig. 2F, *P* < 0.01, by t-test), accompanied by an increase in liver weight (Fig. 2F, *P* < 0.001, by t-test). Histological analysis of liver metastatic lesions by hematoxylin and eosin (H&E) staining further confirmed that the COP1-OE group exhibited more metastatic nodules than the Vector group. We next performed IHC staining of the metastatic nodules using COP1 and the proliferation marker MKI67 (Fig. 2G). The results showed that COP1 expression was significantly higher in metastatic lesions from the COP1-OE group compared with the Vector group, confirming the successful establishment of stable overexpression models. Moreover, MKI67 expression was significantly increased in the metastatic nodules of the COP1-OE group, indicating enhanced proliferative activity in liver metastatic lesions upon COP1 overexpression.

To further validate these findings, we performed additional experiments in immunocompetent wild-type mice. As shown in Fig. 2H, Cop1 was stably overexpressed in the murine CRC cell line MC38. In vitro functional assays demonstrated that Cop1-OE significantly enhanced the invasive capacity of MC38 cells (Fig. 2I, *P* < 0.01, by t-test). In vivo bioluminescence imaging further revealed that Cop1-OE MC38 cells exhibited a significantly increased propensity to metastasize to the liver (Fig. 2J, *P* < 0.01, by t-test). Consistent with these observations, subsequent anatomical analysis showed that mice in the Cop1-OE group developed a greater number of liver metastatic nodules (Fig. 2K, *P* < 0.01, by t-test), accompanied by a significant increase in liver weight (Fig. 2K, *P* < 0.001, by t-test). Similarly, IHC analysis of liver metastatic lesions indicated that Cop1 overexpression was accompanied by elevated Mki67 expression, suggesting enhanced proliferative activity in Cop1-driven metastatic tumors (Fig. 2L).

### COP1 interacts with LUZP1

**Fig. 3:**
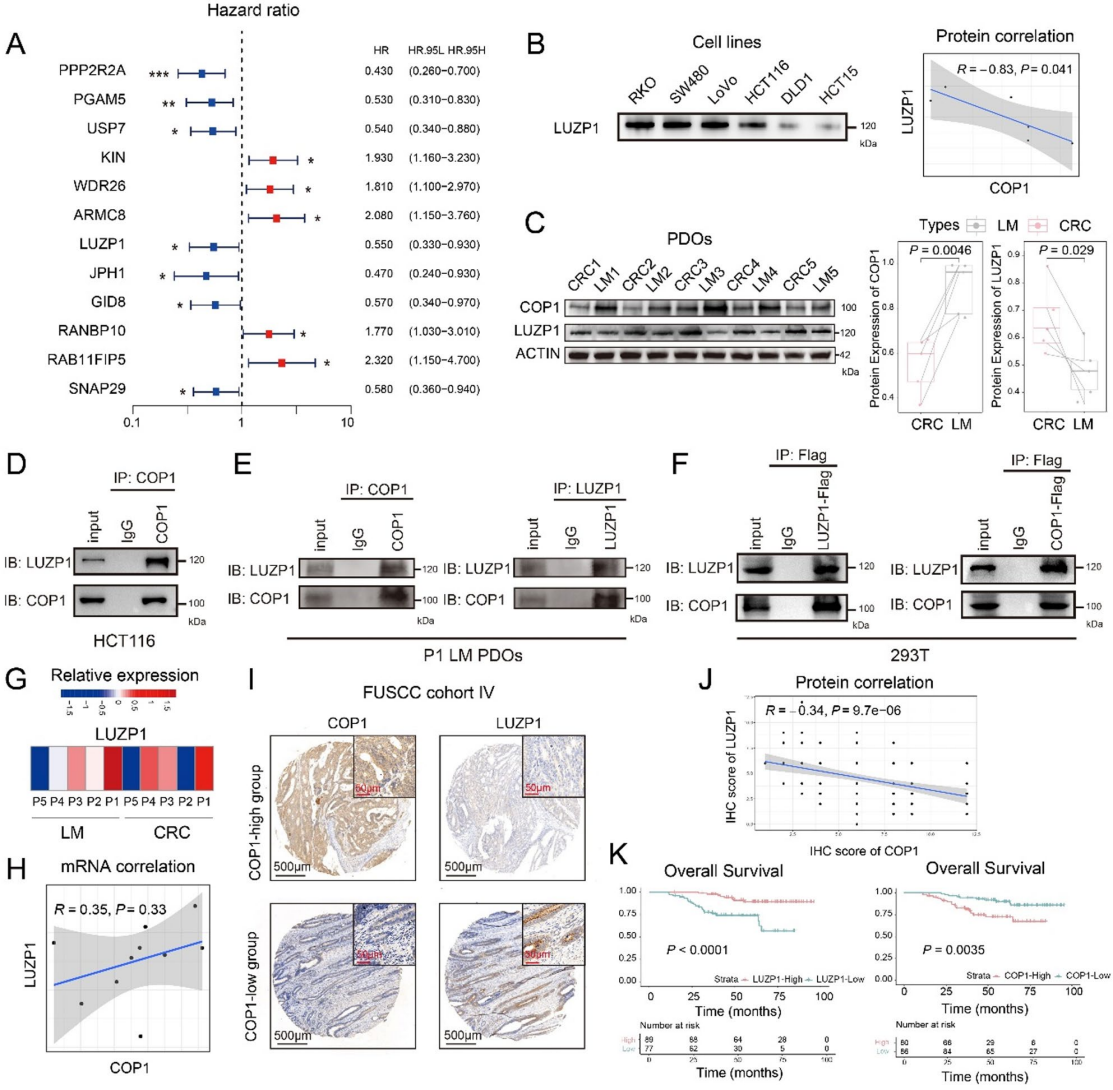
COP1 interacts with LUZP1, and LUZP1 expression positively correlates with colorectal cancer patient survival. **A** A forest plot showing CRC prognosis-associated genes with statistically significant impact among the top 50 COP1-interacting candidates identified by IP-MS analysis. **B** Western blot analysis showing LUZP1 protein expression levels across different human CRC cell lines. The actin loading control from Fig. 2A was used as the reference, as the immunoblots were generated in the same experimental batch. Associations were assessed using Pearson correlation analysis. **C** Immunoblot analysis of COP1 and LUZP1 expression in paired CRLM PDOs from FUSCC Cohort. Boxplots showing quantification of COP1 and LUZP1 protein expression levels in CRC (*n* = 5) and LM (*n* = 5) PDOs. Each line connects paired samples from the same patient. Data were presented as mean ± SD, with individual data points shown; t-test. **D** An interaction assay was performed to evaluate the endogenous binding between COP1 and LUZP1 in HCT116 cells, which endogenously express high levels of both proteins, were used for co-immunoprecipitation (co-IP). **E** An interaction assay was performed to evaluate the endogenous binding between COP1 and LUZP1 in P1 LM PDOs, which endogenously express high levels of both proteins, were used for co-IP. Whole cell lysates were immunoprecipitated with an anti-COP1 antibody and subsequently analyzed by immunoblotting with antibodies against COP1 and LUZP1. **F** Right: An interaction assay was conducted to assess the binding between COP1 and LUZP1. 293T cells were transfected with expression plasmids for LUZP1-Flag. Cell lysates were subsequently subjected to immunoprecipitation using an anti-Flag antibody, followed by immunoblotting with antibodies against both COP1 and LUZP1. Left: An interaction assay was conducted to assess the binding between COP1 and LUZP1. 293T cells were transfected with expression plasmids for COP1-Flag. Cell lysates were subsequently subjected to immunoprecipitation using an anti-Flag antibody, followed by immunoblotting with antibodies against both COP1 and LUZP1. **G** A heatmap was generated to display the RNA expression levels of LUZP1 in paired CRLM organoids. **H** A scatter plot illustrating the correlation between LUZP1 and COP1 RNA expression levels. Associations were assessed using Pearson correlation analysis. **I** IHC showing COP1 and LUZP1 protein levels in TMA from CRC primary lesions in FUSCC Cohort IV; scale bar = 500μm. **J** A scatter plot illustrating the correlation between LUZP1 and COP1 protein expression levels (IHC score) in TMA of FUSCC Cohort IV. Associations were assessed using Pearson correlation analysis. **J** Patients from FUSCC Cohort IV were stratified into high (IRS > 6) and low expression groups based on IHC score. Box plots demonstrated that CRC patients with high COP1 protein expression exhibited significantly lower levels of LUZP1 protein, while those with high LUZP1 protein expression showed reduced COP1 protein levels; t-test. **K** Kaplan-Meier Plot showing OS of CRC patients (FUSCC Cohort IV) stratified by COP1 and LUZP1 expression levels; log-rank test. * *P* < 0.05, ** *P* < 0.01, *** *P* < 0.001

We performed immunoprecipitation coupled with mass spectrometry (IP-MS) analysis to identify COP1-interacting proteins (Supplementary Table S3). A prognostic analysis of the top 50 candidates was conducted using the TCGA database. The results revealed that several proteins, including PPP2R2A, PGAM5, USP7, LUZP1, JPH1, GID8, and SNAP29, were significantly associated with better prognosis in CRC (Fig. 3A). Western blot (WB)-based correlation analysis across multiple CRC cell lines identified LUZP1 as a candidate protein exhibiting an inverse correlation with COP1 expression (Fig. 3B, *R* = -0.83, *P* = 0.041, by Pearson correlation analysis). Note that the immunoblots in Fig. 3B were processed in the same batch as those in Fig. 2A; thus, the actin loading control from Fig. 2A was used for normalization in Fig. 3B. Protein-level validation in PDOs from FUSCC Cohort I confirmed increased COP1 expression in LM organoids compared with CRC organoids (Fig. 3C, *P* = 0.0046, by t-test), while LUZP1 showed an inverse expression pattern (Fig. 3C, *P* = 0.029, by t-test). We next selected HCT116 cells exhibiting moderate endogenous expression of both COP1 and LUZP1 to perform endogenous co-immunoprecipitation (co-IP) assays (Fig. 3D). In parallel, P1-LM PDOs expressing both COP1 and LUZP1 were similarly analyzed by endogenous co-IP (Fig. 3E). The results indicated that COP1 and LUZP1 interact under endogenous conditions. Exogenous overexpression of LUZP1 or COP1 in 293T cells also confirmed their interaction in co-IP assays (Fig. 3F). Finally, immunofluorescence co-staining revealed cytoplasmic co-localization of COP1 and LUZP1 (Figure S3A), supporting their direct intracellular interaction.

As an E3 ubiquitin ligase, COP1 regulates protein stability at the post-translational level. RNA sequencing of organoids revealed no negative correlation between COP1 and LUZP1 mRNA expression levels at the transcriptional level (Fig. 3G and H). However, IHC analysis of a primary tumor TMA (FUSCC Cohort IV) revealed a significant negative correlation between COP1 and LUZP1 protein expression (Fig. 3I), with a correlation coefficient of -0.34 (Fig. 3J, *P* = 9.7e-06, by Pearson correlation analysis). We first examined the associations between COP1 and LUZP1 expression-based stratification and clinicopathological characteristics of FUSCC Cohort IV. We observed that the proportion of patients with normal CEA levels was higher in the high LUZP1 expression group than in the low LUZP1 expression group (Table 3). Kaplan-Meier survival analysis further demonstrated that, in patients with primary CRC tumor, high COP1 expression was significantly associated with poor prognosis (OS, *P* = 0.0035, by log-rank test), whereas high LUZP1 expression was significantly correlated with favorable survival outcomes (OS, *P* < 0.0001, log-rank test) (Fig. 3K). In patients with CRLM of FUSCC III, elevated LUZP1 expression likewise predicted improved prognosis (OS, *P* < 0.0001, by log-rank test; DFS, *P* = 0.0017, by log-rank test), exhibiting an opposite prognostic trend to that of COP1 expression (Fig. 1K and Figure S1F).

**Table 3:** Clinical feature analysis based on COP1 and LUZP1 expression groups in CRC-TMA (FUSCC IV)

Characteristics	COP1			LUZP1		
	High expression group (*N* = 80), %	Low expression group (*N* = 86), %	*P* value	High expression group (*N* = 89), %	Low expression group (*N* = 77), %	*P* value
Age			0.5343			> 0.9999
≤ 60	41 (51.25)	39 (45.35)		43 (48.31)	37 (48.05)	
> 60	39 (48.75)	47 (54.65)		46 (51.69)	40 (51.95)	
Gender			0.2109			> 0.9999
Male	50 (62.50)	45 (52.33)		51 (57.30)	44 (57.14)	
Female	30 (37.50)	41 (47.67)		38 (42.70)	33 (42.86)	
Primary site			0.5539			0.7565
Colon	40 (50.00)	48 (55.81)		46 (51.69)	42 (54.55)	
Rectum	40 (50.00)	38 (44.19)		43 (48.31)	35 (45.45)	
CEA			0.4073			0.0321
Normal	51 (63.75)	61 (70.93)		67 (75.28)	45 (58.44)	
Abnormal	29 (36.25)	25 (29.07)		22 (24.72)	32 (41.56)	
pT stage			0.8671			> 0.9999
3	54 (67.50)	60 (69.77)		61 (68.54)	53 (68.83)	
4	26 (32.50)	26 (30.23)		28 (31.46)	24 (31.17)	
Histological type			0.4364			0.2921
Adenocarcinoma	74 (92.50)	76 (88.37)		78 (87.64)	72 (93.51)	
Mucinous/SRCC	6 (7.50)	10 (11.63)		11 (12.36)	5 (6.49)	
Differentiation			> 0.9999			> 0.9999
Moderately-poorly	3 (3.75)	3 (3.49)		3 (3.37)	3 (3.90)	
Highly-moderately	77 (96.25)	83 (96.51)		86 (96.63)	74 (96.10)	
Lymphovascular invasion			0.7760			0.0906
Yes	73 (91.25)	80 (93.02)		79 (88.76)	74 (96.10)	
No	7 (8.75)	6 (6.98)		10 (11.24)	3 (3.90)	
Perineural invasion			> 0.9999			0.6980
Yes	64 (80.00)	69 (80.23)		70 (78.65)	63 (81.82)	
No	16 (20.00)	17 (19.77)		19 (21.35)	14 (18.18)	
KRAS mutation			0.1451			0.2266
Yes	31 (38.75)	28 (32.56)		29 (32.58)	30 (38.96)	
No	36 (45.00)	33 (38.37)		35 (39.33)	34 (44.16)	
Not tested	13 (16.25)	25 (29.07)		25 (28.09)	13 (16.88)	
BRAF^**V600E**^ mutation			0.1218			0.0788
Yes	5 (6.25)	3 (3.49)		2 (2.25)	6 (7.79)	
No	62 (77.50)	58 (67.44)		62 (69.66)	58 (75.33)	
Not tested	13 (16.25)	25 (29.07)		25 (28.09)	13 (16.88)	
MSI status			0.0785			0.2190
Yes	4 (5.00)	7 (8.14)		5 (5.62)	6 (7.79)	
No	63 (78.75)	54 (62.79)		59 (66.29)	58 (75.32)	
Not tested	13 (16.25)	25 (29.07)		25 (28.09)	13 (16.89)	

### LUZP1 inhibits colorectal cancer growth and invasiveness in vitro and in vivo

**Fig. 4:**
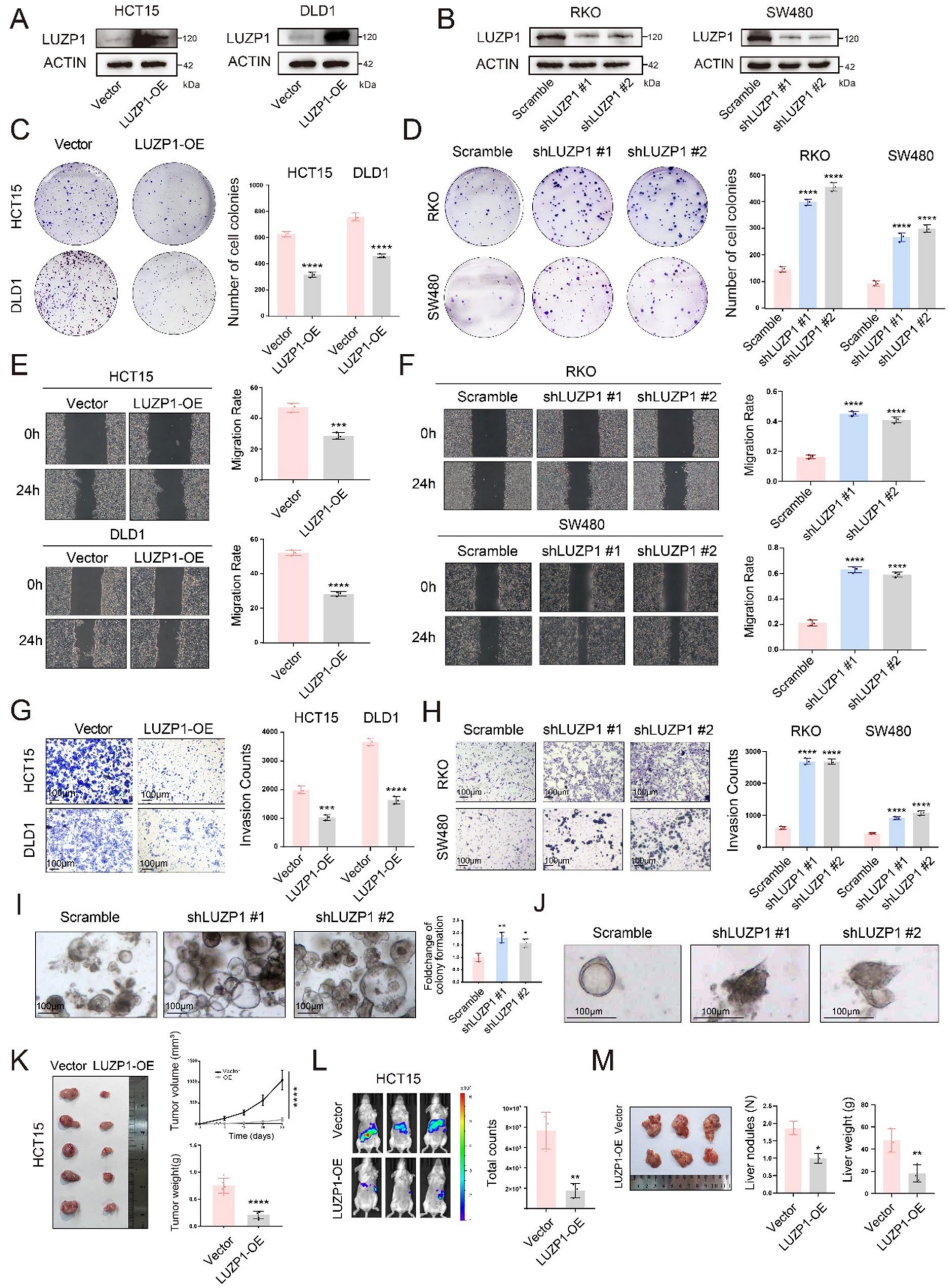
LUZP1 inhibits colorectal cancer growth and invasiveness in vitro and in vivo. **A** Immunoblot validation of stable LUZP1-overexpressing (LUZP1-OE) HCT15 and DLD1 cell lines. **B** Immunoblot validation of stable LUZP1 knockdown (LUZP1-KD) in RKO and SW480 cell lines. **C** Colony formation assays were performed to compare the proliferative capacity of LUZP1-overexpressing HCT15 and DLD1 cells with their respective vector control groups (each group *n* = 3); t-test. **D** Colony formation assays were performed to compare the proliferative capacity of LUZP1 knockdown RKO and SW480 cells with their respective negative control (NC) groups (each group *n* = 3); t-test. **E** Wound healing assays were conducted to evaluate the migratory ability of LUZP1-overexpressing HCT15 and DLD1 cells compared to their respective vector control groups (each group *n* = 3); t-test. **F** Wound healing assays were conducted to evaluate the migratory ability of LUZP1 knockdown RKO and SW480 cells with their respective negative control (NC) groups (each group *n* = 3); t-test. **G** Invasion assays were conducted to assess the invasive capacity of LUZP1-overexpressing HCT15 and DLD1 cells in comparison with their respective vector control groups (each group *n* = 3); scale bar = 100μm; t-test. **H** Invasion assays were conducted to assess the invasive capacity of LUZP1 knockdown RKO and SW480 cells with their respective negative control (NC) groups (each group *n* = 3); scale bar = 100μm; t-test. **I** Representative bright-field images of colorectal cancer PDOs transduced with scramble control or two independent shRNAs targeting LUZP1 (shLUZP1 #1 and #2). Right, quantification of organoid formation capacity, shown as fold change relative to scramble control. Scale bar = 100μm. **J** Representative images showing the invasive morphology of PDOs following LUZP1 knockdown using two independent shRNAs. Scale bar = 100μm. **K** In vivo transplantation experiments were performed to compare the tumor growth capacity of LUZP1-overexpressing DLD1 cells with that of vector control cells; t-test. **L** In vivo bioluminescence imaging of mice bearing HCT15 xenografts with vector or LUZP1-OE cells. Right, quantification of total photon counts; t-test. **M** Representative images of liver metastatic nodules derived from vector control or LUZP1-OE HCT15 cells (each group *n* = 3), with quantification of liver nodule number and liver weight; t-test. Data are presented as mean ± SD, with individual data points shown. * *P* < 0.05, ** *P* < 0.01, *** *P* < 0.001, **** *P* < 0.0001

To further elucidate the role of LUZP1 in CRC, we first conducted colony formation assays using the corresponding stably transduced cell lines (Fig. 4A and B), showing that LUZP1 suppresses tumor cell proliferation (Fig. 4C and D). Wound healing assays further revealed that LUZP1 inhibited cell migration (Fig. 4E and F), while invasion assays confirmed its suppressive effect on tumor cell invasiveness (Fig. 4G and H). Collectively, these results indicated that LUZP1 markedly impairs the migratory and invasive capabilities of CRC cells in vitro.

To assess the potential impact of altered LUZP1 expression on organoid behavior, we performed LUZP1-knockdown in organoid models and compared the results with control organoids. LUZP1-knockdown led to an increase in organoid numbers (Fig. 4I). In addition, guided by previously published protocols [24], we conducted organoid invasion assays and noted that LUZP1-knockdown organoids developed protrusive leading fronts (Fig. 4J). These findings suggested that LUZP1 expression may constrain organoid proliferation and invasive capacity, a trend that was consistent with our observations in cell line-based experiments.

To evaluate the effects of LUZP1 in vivo, we performed xenograft experiments in nude mice using a stable LUZP1-overexpressing (LUZP1-OE) HCT15 cell line. LUZP1 overexpression significantly inhibited tumor growth, as evidenced by a substantial reduction in tumor volume and weight in LUZP1-OE groups relative to controls (Fig. 4K). Conversely, LUZP1-knockdown significantly accelerated tumor growth, with both tumor volume and weight notably increased in knockdown groups compared to controls (Figure S3B-S3C). In a splenic injection liver metastasis model, LUZP1-OE significantly impaired the hepatic colonization ability of CRC cells, as measured by bioluminescent signal (Fig. 4L) and metastatic burden (Fig. 4M). These results functionally link LUZP1 loss to enhanced metastatic potential in vivo.

### COP1 promotes LUZP1 ubiquitination and degradation

**Fig. 5:**
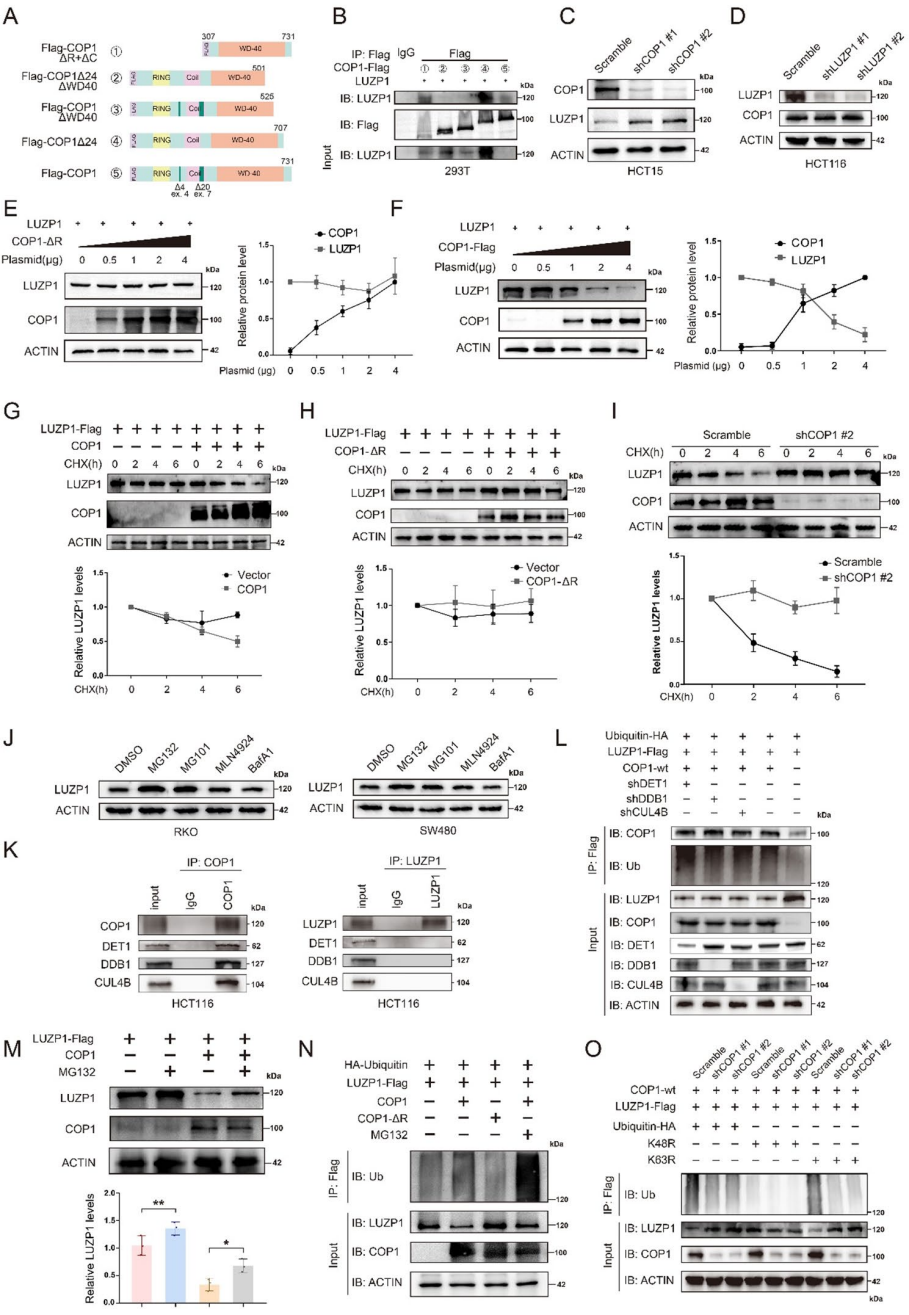
COP1 promotes LUZP1 ubiquitination and degradation. **A** A schematic diagram illustrating the different truncated variants of the COP1 sequence within the Flag-COP1 constructs. **B** An interaction assay was conducted to evaluate the binding between LUZP1 and five different Flag-tagged COP1 variants introduced exogenously. 293T cells were co-transfected with expression plasmids encoding LUZP1 and each of Flag-COP1 variants. Cell lysates were subjected to immunoprecipitation using an anti-Flag antibody, followed by immunoblotting with anti-Flag and anti-LUZP1 antibodies to assess their interaction. **C** Western blot was performed to evaluate changes in LUZP1 expression levels following COP1 knockdown in HCT15 cells. **D** Immunoblot analysis examining the effect of RNAi-mediated LUZP1 depletion on COP1 protein expression in HCT116 cells. **E** Western blot was conducted to examine LUZP1 protein expression levels following overexpression of a COP1 variant lacking RING domain (COP1-ΔRING) at increasing plasmid doses. **F** Western blot was conducted to examine LUZP1 protein expression levels following overexpression of a full-length COP1 (COP1-Flag) at increasing plasmid doses. **G-H** 293T cells were transfected with plasmids expressing Flag-tagged LUZP1 along with either full-length COP1 or the RING domain–deleted mutant (COP1-ΔR). After treatment with 20 µg/ml cycloheximide (CHX), cells were collected at specified time points. Protein levels of LUZP1 and COP1 in the lysates were then analyzed by Western blotting. **I** Cells were transfected with either control scramble RNA or COP1-targeting shRNA #2, followed by treatment with 20 µg/ml cycloheximide (CHX). Samples were harvested at designated time intervals, and protein expression was analyzed by immunoblotting using the specified antibodies. **J** Western blot was performed to assess LUZP1 protein levels in RKO and SW480 cells with high endogenous LUZP1 expression following treatment with different inhibitors. **K** co-IP analysis showing the interaction of COP1 or LUZP1 with components of the CRL4 components (DET1, DDB1, and CUL4B) in 293T cells. Cell lysates were subjected to immunoprecipitation using anti-COP1 or anti-LUZP1 antibodies, followed by immunoblotting for DET1, DDB1, and CUL4B. **L** co-IP analysis assessing LUZP1 ubiquitination in the context of COP1 expression and the CRL4 complex (DET1, DDB1, and CUL4B) depletion in 293T cells. HA-ubiquitin and FLAG-LUZP1 were co-expressed, with or without COP1-wt and shRNAs targeting DET1, DDB1, or CUL4B. **M** Cells transfected with either LUZP1-Flag or COP1 were treated with 10µM MG132 or left untreated for 8 h, followed by collection and immunoblotting with the specified antibodies. A corresponding graph shows the quantification of LUZP1 protein levels, normalized to actin (each group *n* = 3). **N** Immunoprecipitated exogenous LUZP1 from 293T cells transfected with the specified plasmids was analyzed by immunoblotting using an anti-ubiquitin antibody. **O** Analysis of LUZP1 ubiquitination linkage specificity. 293T cells were co-transfected with HA-ubiquitin, FLAG-LUZP1, and wild-type COP1, together with K48R or K63R ubiquitin mutants, in the presence or absence of COP1-targeting shRNAs. * *P* < 0.05, ** *P* < 0.01

To investigate the structural basis of the COP1-LUZP1 interaction, we constructed five different FLAG-tagged COP1 truncation mutants (Fig. 5A). Overexpression of the 425AA (①) mutant together with LUZP1 allowed detection of both proteins, indicating that the WD40 domain of COP1 mediates its interaction with LUZP1. However, due to the lack of the RING domain and coiled-coil region, LUZP1 protein was not degraded in this context. In contrast, when LUZP1 was co-expressed with 501AA (②) or 525AA mutants (③)—both lacking part of the WD40 domain—LUZP1 protein could not be detected, suggesting that an intact WD40 domain is required for the COP1-LUZP1 interaction. Both 707AA (④) and full-length 731AA (⑤) mutants could bind to LUZP1. Notably, overexpression of 731AA (⑤) led to a marked reduction in LUZP1 protein levels, while 707AA (④), which exhibited a dominant-negative effect, was unable to mediate LUZP1 degradation despite successful binding (Fig. 5B). Thus, COP1 binds to LUZP1 via its complete WD40 domain and promote LUZP1 protein degradation through its RING domain and coiled-coil region. Consistent with these experimental findings, protein-protein docking using GRAMM predicted that the WD40 domain of COP1 binds to LUZP1, with hydrogen bond distances of 1.5–3.5Å and a favorable binding free energy (Δ^!^G = -24.9 kcal/mol) calculated by PDBePISA, indicating a strong and stable COP1-LUZP1 interaction (Figure S3D).

To validate that COP1 mediates LUZP1 degradation through its RING domain, we first knocked down COP1 and observed a corresponding increase in LUZP1 protein levels (Fig. 5C). We also assessed whether modulating LUZP1 levels reciprocally affects COP1. Western blot analysis showed that neither LUZP1 knockdown (Fig. 5D) nor overexpression (Figure S3E) altered COP1 protein abundance, indicating that COP1 acts upstream of LUZP1 within this regulatory pathway. Next, dose-dependent overexpression of a COP1 construct lacking the RING domain (COP1-ΔRING) did not significantly change in LUZP1 protein levels (Fig. 5E). In contrast, dose-dependent overexpression of full-length COP1 led to a gradual decrease in LUZP1 protein levels (Fig. 5F). We then inhibited protein synthesis using cycloheximide (CHX). In the presence of full-length COP1, LUZP1 degradation was markedly accelerated (Fig. 5G). However, this effect was absent when COP1-ΔRING was overexpressed (Fig. 5H). Moreover, under CHX treatment, COP1-knockdown resulted in sustained high levels of LUZP1 protein compared to scramble controls (Fig. 5I). These findings collectively demonstrated that COP1 could bind to LUZP1 and promote its degradation via its RING domain.

To determine whether LUZP1 degradation occurs through ubiquitination, we first examined the effects of various inhibitors on endogenous LUZP1 levels. Proteasome inhibitors [28] (MG132 and MG101) caused accumulation of LUZP1, while neither the neddylation inhibitor MLN4924 [29] nor the lysosomal inhibitor BafA1 [30] showed this effect (Fig. 5J). To directly assess whether the canonical DET1-DDB1-CUL4 (CRL4) complex is required for the COP1-mediated LUZP1 degradation, we first performed a co-IP assay which confirmed that COP1 associates with the core CRL4 components (DET1, DDB1, and CUL4B) (Fig. 5K). A separate co-IP assay revealed that LUZP1 does not interact with any of these CRL4 components (DET1, DDB1, and CUL4B) (Fig. 5K). We then individually knocked down each core CRL4 components (DET1, DDB1, and CUL4B) in COP1-overexpressing cells. Notably, COP1 remained capable of effectively reducing LUZP1 protein levels in the absence of each CRL4 component (Fig. 5L). These genetic data indicated that COP1-mediated degradation of LUZP1 occurs independently of the CRL4 machinery, thereby distinguishing this mechanism from previously reported CRL4-dependent functions of COP1.

Our western blot analyses consistently showed that COP1-mediated regulation of LUZP1 protein levels does not rely on CRL activity, suggesting that COP1 may directly ubiquitinate LUZP1 to control its stability. This was further supported by the observation that MG132 treatment effectively prevented COP1-induced LUZP1 degradation, indicating that COP1 targets LUZP1 for proteasomal degradation (Fig. 5M). Moreover, in the absence of MG132, full-length COP1 induced pronounced degradation of LUZP1 along with a noticeable increase in its ubiquitination, whereas the RING domain-deleted mutant (COP1-ΔRING) exhibited a weaker effect (Fig. 5N). In contrast, upon MG132 treatment, COP1 significantly enhanced LUZP1 ubiquitination, but its degradation was attenuated (Fig. 5N and S3F). Collectively, these findings indicated that COP1 promotes ubiquitination and subsequent proteasomal degradation of LUZP1 in a RING domain-dependent manner.

To further define the biochemical nature of this degradation signal, we performed ubiquitination assays using lysine-specific ubiquitin mutants. Co-expression of a K48R ubiquitin mutant (which abrogates K48-linked polyubiquitin chains) significantly reduced COP1-induced LUZP1 ubiquitination, whereas a K63R mutant had minimal effect (Fig. 5O). These results demonstrated that COP1 primarily promotes K48-linked polyubiquitination of LUZP1, the canonical signal for proteasomal targeting, consistent with the observed decrease in LUZP1 stability.

### COP1 promotes EMT activation by degrading LUZP1 and enhancing DAPK3-mediated MYL9 phosphorylation

To explore the mechanism by which COP1 promotes CRLM through the ubiquitin-mediated degradation of LUZP1, we first performed functional enrichment analysis of RNA-seq data from PDOs (Figure S2B). This analysis revealed that PDOs with low LUZP1 expression exhibited features of mesenchymal transition, indicating activation of the epithelial-mesenchymal transition (EMT) pathway. Previous studies have shown that LUZP1 can interact with DAPK3 and MYL9, inhibiting DAPK3-mediated phosphorylation of MYL9—a modification known to enhance tumor cell metastasis [31]. We therefore hypothesized that COP1-mediated degradation of LUZP1 might relieve its inhibitory effect on DAPK3, thereby promoting MYL9 phosphorylation and EMT activation.

**Fig. 6:**
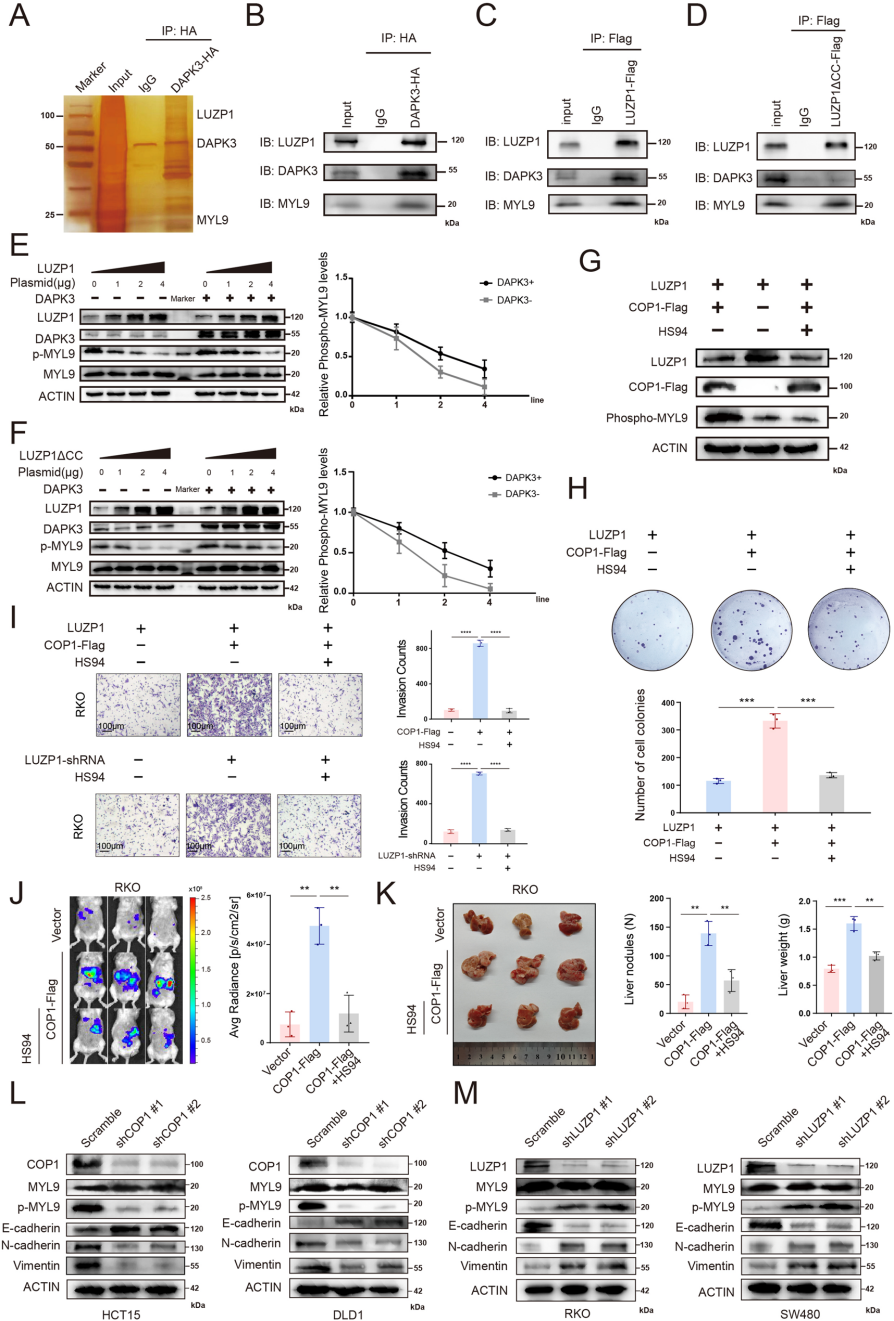
COP1 promotes EMT activation by degrading LUZP1 and enhancing DAPK3-mediated MYL9 phosphorylation. **A** 293T cells were transfected with exogenous DAPK3-HA, and co-immunoprecipitation was performed using an anti-HA antibody to identify DAPK3-interacting proteins. **B** An interaction assay was conducted to assess the binding between DAPK3 and LUZP1. 293T cells were transfected with expression plasmids for DAPK3-HA. Cell lysates were subsequently subjected to immunoprecipitation using an anti-HA antibody, followed by immunoblotting with antibodies against both DAPK3 and LUZP1. **C** An interaction assay was conducted to assess the binding between DAPK3 and LUZP1. 293T cells were transfected with expression plasmids for LUZP1-Flag. Cell lysates were subsequently subjected to immunoprecipitation using an anti-Flag antibody, followed by immunoblotting with antibodies against both DAPK3 and LUZP1. **D** An interaction assay was conducted to assess the binding between DAPK3 and LUZP1. 293T cells were transfected with expression plasmids for LUZP1△cc-Flag. Cell lysates were subsequently subjected to immunoprecipitation using an anti-Flag antibody, followed by immunoblotting with antibodies against both DAPK3 and LUZP1. **E** 293T cells were transfected with expression plasmids for DAPK3 and LUZP1. Western blot analysis was then performed to evaluate the protein levels of MYL9 and phosphorylated MYL9 (p-MYL9) downstream of DAPK3. **F** 293T cells were transfected with expression plasmids for DAPK3 and LUZP1△cc-Flag. Western blot analysis was then performed to evaluate the protein levels of MYL9 and phosphorylated MYL9 (p-MYL9) downstream of DAPK3. **G** In 293T cells with exogenous overexpression of LUZP1, COP1 was further overexpressed to assess its effect on MYL9 phosphorylation. Cells were also treated with HS94 (a DAPK3 inhibitor, 20µM), and phosphorylated MYL9 levels were evaluated by Western blot. **H** Cells were treated as described in **G**, and colony formation assays were performed to assess the proliferative capacity across different groups (each group *n* = 3). **I** Invasion assays were conducted to evaluate the invasive capacity of cells across different experimental groups (each group *n* = 3). **J** In vivo bioluminescence imaging of mice bearing RKO liver metastasis models established with vector control or COP1-FLAG–overexpressing cells, in the presence or absence of the DAPK3 inhibitor HS94. Left, representative bioluminescence images; right, quantification of average radiance (each group *n* = 3). **K** Representative images of LM nodules from the indicated groups. Right, quantification of LM number and liver weight; t-test; **L.** Western blot analysis was performed following COP1 knockdown to assess the expression levels of downstream MYL9, phosphorylated MYL9 (p-MYL9), and EMT-related proteins; scale bar = 100 μm. **M** Western blot was performed following LUZP1 knockdown to assess the expression levels of downstream MYL9, phosphorylated MYL9 (p-MYL9), and EMT-related proteins. Data are presented as mean ± SD. * *P* < 0.05, ** *P* < 0.01, *** *P* < 0.001, **** *P* < 0.0001

Silver staining experiments suggested that DAPK3 could interact with both LUZP1 and MYL9 (Fig. 6A). Co-IP assays confirmed the interaction among LUZP1, DAPK3, and MYL9 (Fig. 6B and C). Hyodo et al. demonstrated that, when the coiled-coil (cc) domain of LUZP1 was truncated, LUZP1 lost its ability to bind DAPK3, while still retaining its interaction with MYL9 and its inhibitory effect on DAPK3-mediated phosphorylation of MYL9 [31]. Accordingly, we constructed a plasmid expressing a coiled-coil domain-truncated form of LUZP1 (LUZP1△cc-Flag), and further confirmed via co-IP that LUZP1△cc-Flag could no longer bind to DAPK3 but still interacted with MYL9 (Fig. 6D). Both the full-length LUZP1 and LUZP1△cc-Flag were able to inhibit MYL9 phosphorylation, while overexpression of DAPK3 partially restored p-MYL9 levels (Fig. 6E and F).

To further validate the relationship among COP1, LUZP1, and MYL9, we overexpressed COP1 in the presence of LUZP1 and observed that degradation of LUZP1 was accompanied by an increase in phosphorylated MYL9 (p-MYL9) levels (Fig. 6G). Upon treatment with the DAPK3 inhibitor HS94 [26], p-MYL9 levels were reduced, indicating that DAPK3 activity is responsible for MYL9 phosphorylation (Fig. 6G). Colony formation assays demonstrated that COP1-mediated degradation of LUZP1 promoted CRC cell growth, which was significantly suppressed by HS94 (Fig. 6H). Transwell assays further showed that the enhanced invasive capacity induced by COP1 overexpression or LUZP1 knockdown could be suppressed by HS94 treatment (Fig. 6I). We also performed genetic rescue experiments via DAPK3-knockdown. In COP1-overexpressing cells, the enhanced proliferative and invasive phenotypes were significantly attenuated upon DAPK3-knockdown (Figure S4A-S4B).

Most importantly, we tested the functional relevance of this axis in vivo in a liver metastasis model. As shown in Fig. 2, COP1 overexpression in RKO cells dramatically increased hepatic metastatic burden. Crucially, co-treatment with the DAPK3 inhibitor HS94 in this model significantly reduced both the number of metastatic nodules and liver weight in COP1-overexpressing mice (Fig. 6J and K). These results demonstrated that pharmacological inhibition of DAPK3 can effectively reverse COP1-driven metastasis in vivo, providing compelling preclinical evidence for the therapeutic potential of targeting this node. Together, these findings suggested that the pro-tumorigenic and pro-metastatic effects of COP1 may be mediated through the COP1-LUZP1-DAPK3-MYL9 axis.

Furthermore, COP1-knockdown in HCT15 and DLD1 cells led to a marked reduction in p-MYL9 levels and was accompanied by coordinated changes in key EMT markers, characterized by increased expression of the epithelial marker E-cadherin and decreased expression of the mesenchymal markers N-cadherin and Vimentin (Fig. 6L). Conversely, COP1-overexpression in RKO and SW480 cells induced the opposite expression pattern (Figure S4C). Moreover, DAPK3-knockdown based rescue experiments in COP1-overexpressing cells demonstrated that silencing DAPK3 effectively abrogated COP1-induced EMT activation. Similarly, knockdown of LUZP1 in RKO and SW480 cells led to a significant increase in p-MYL9 levels and a corresponding EMT phenotype, characterized by downregulation of E-cadherin and upregulation of N-cadherin and Vimentin (Fig. 6M). Overexpression of LUZP1 in HCT15 and DLD1 cells reversed these changes (Figure S4D). Finally, extending the COP1 dose-escalation experiment (Fig. 5F) to downstream effectors revealed that increasing COP1 expression induced a dose-dependent reduction in LUZP1, accompanied by increased p-MYL9 and Vimentin levels (Figure S5). These findings collectively suggested that COP1-mediated degradation of LUZP1 is functionally linked to elevated MYL9 phosphorylation and is accompanied by EMT-associated features and increased metastatic potential of CRC cells.

### COP1 promotes oxaliplatin resistance in colorectal cancer

**Fig. 7:**
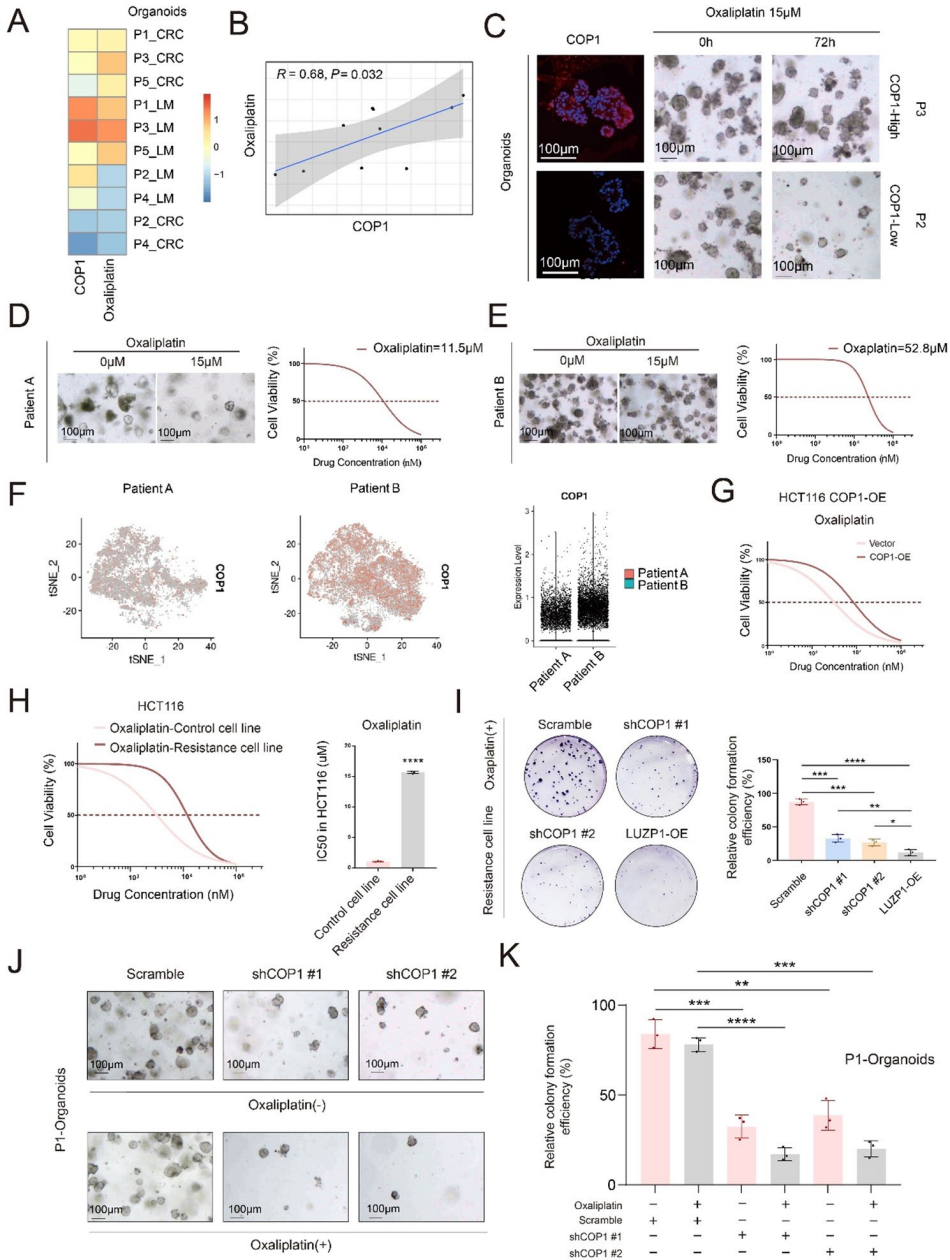
COP1 promotes oxaliplatin resistance in colorectal cancer. **A** A heatmap was generated to display the expression levels of COP1 in PDOs alongside the IC50 values for oxaliplatin. The oxaliplatin IC₅₀ in our PDO panel was evaluated using Pearson correlation analysis. **B** Scatter plots were generated to illustrate the correlation between COP1 expression levels and the IC50 values of oxaliplatin. **C** Bright-field microscopy images show the viability of high-COP1-expressing P3-derived and low-COP1-expressing P2-derived organoids after treatment with 15µM oxaliplatin; scale bar = 100μm. **D** Organoids derived from Patient A exhibited an oxaliplatin IC50 of 11.5µM. Bright-field microscopy images show the viability of these PDOs following treatment with 15µM oxaliplatin; scale bar = 100μm. The data points shown in the dose-response analyses represent the mean ± SD of three technical replicates. Due to minimal variance across technical replicates, individual data points overlapped extensively; therefore, to maintain visual clarity in the figures, only the mean values are plotted, and a single best-fit curve is displayed for Patient A PDOs. **E** Organoids derived from Patient B exhibited an oxaliplatin IC50 of 52.8µM; scale bar = 100μm. Bright-field microscopy images show the viability of these PDOs following treatment with 15µM oxaliplatin. The data points shown in the dose-response analyses represent the mean ± SD of three technical replicates. Due to minimal variance across technical replicates, individual data points overlapped extensively; therefore, to maintain visual clarity in the figures, only the mean values are plotted, and a single best-fit curve is displayed for Patient B PDOs. **F** Single-cell analysis was performed to compare COP1 expression levels in PDOs derived from Patient A and Patient B. **G** IC50 assays were performed to evaluate the sensitivity of HCT116 COP1-overexpressing cell lines to oxaliplatin and. **H** The IC50 values of oxaliplatin were compared between the HCT116 oxaliplatin-resistant cell line and the control group; t-test. The data points shown in the dose-response analyses represent the mean ± SD of these technical replicates. Due to minimal variance across technical replicates, individual data points overlapped extensively; therefore, to maintain visual clarity in the figures, only the mean values are plotted, and a single best-fit curve is displayed for Patient A PDOs. **I** Cell proliferation assays were performed to evaluate the growth capacity of HCT116 oxaliplatin-resistant cells following COP1 knockdown or LUZP1 overexpression (each group *n* = 3); t-test. **J**–**K**. In PDOs derived from Patient P1, which exhibited a high IC50 for oxaliplatin, COP1 was knocked down, and cell proliferation was assessed with or without oxaliplatin treatment (each group *n* = 3); scale bar = 100 μm; t-test. * *P* < 0.05, ** *P* < 0.01, *** *P* < 0.001, **** *P* < 0.0001

Parallel drug sensitivity profiling was conducted on five pairs of CRLM PDOs to evaluate therapeutic responses to oxaliplatin, 5-FU and SN-38 (the active metabolite of irinotecan). The IC50 values for these drugs and COP1 expression levels are summarized in Fig. 7A and B and Figure S6A-S6B (Supplementary Table S4). While COP1 expression was positively correlated with the IC50 of all three agents (all *R* > 0.3), the strongest correlation was observed with oxaliplatin (*R* = 0.68, *P* = 0.032, by Pearson correlation analysis; Fig. 7B), exceeding those for 5-FU (*R* = 0.41, *P* = 0.25, Pearson correlation analysis; Figure S6B) and SN-38 (*R* = 0.39, *P* = 0.26, by Pearson correlation analysis; Figure S6B). Therefore, we focused our subsequent analyses on oxaliplatin sensitivity. As shown in Fig. 7C, PDOs from Patient 3 (P3-PDOs), which exhibited high COP1 expression, showed minimal decrease in organoid number upon oxaliplatin treatment (15µM), whereas PDOs from Patient 2 (P2-PDOs), with low COP1 expression, displayed an obvious reduction. To further examine this relationship, we established two additional CRC PDOs. PDOs from Patient A were sensitive to oxaliplatin (IC50 = 11.5µM, Fig. 7D), whereas PDOs from Patient B were resistant (IC50 = 52.8µM, Fig. 7E). ScRNA-seq of both PDOs revealed that COP1 expression was significantly higher in oxaliplatin-resistant Patient B PDOs compared to sensitive Patient A PDOs (Fig. 7F). These results indicated a strong association between COP1 expression and oxaliplatin resistance in CRC.

Next, we overexpressed COP1 in HCT116 and performed drug sensitivity assays. The results showed that COP1 overexpression conferred a greater resistance to oxaliplatin compared to 5-FU/SN-38 (Fig. 7G and Figure S6C). To directly assess whether COP1 contributed to oxaliplatin resistance, we established an oxaliplatin-resistant HCT116 cell line (Fig. 7H). Knockdown of COP1 in these resistant cells significantly restored sensitivity to oxaliplatin (1µM), as evidenced by reduced colony formation (Fig. 7I). Notably, overexpression of LUZP1 also produced a similar sensitizing effect (Fig. 7I). To validate these findings in a more clinically relevant model, we selected P1-derived CRC organoids, which had IC50 values for oxaliplatin (IC50 = 65.52µM), 5-FU (IC50 = 34.15µM) and SN-38 (IC50 = 1.823µM). Upon COP1 knockdown, P1 organoids exhibited significantly increased sensitivity to oxaliplatin (20µM), resulting in a reduction in organoid number (Fig. 7J and K, Figure S6D-S6E). Together, these results suggested that while COP1 primarily promotes resistance to oxaliplatin-based chemotherapy in colorectal cancer, it may also contribute to a broader chemo-resistant phenotype.

### COP1 induces oxaliplatin resistance by degrading LUZP1 and enhancing DAPK3-mediated MYL9 phosphorylation to activate JAK2-STAT3-CCND2 pathway

**Fig. 8:**
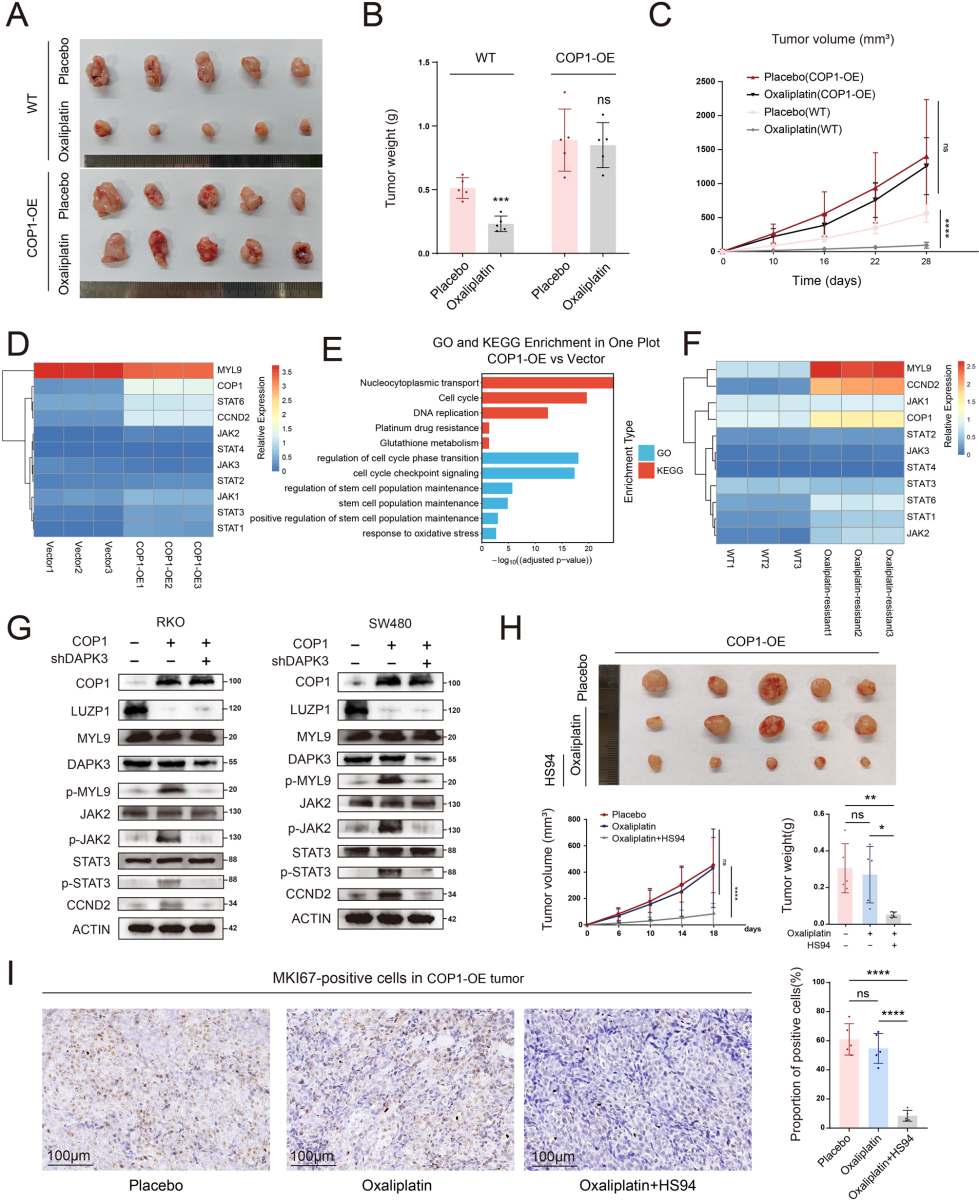
COP1 induces oxaliplatin resistance by degrading LUZP1 and enhancing DAPK3-mediated MYL9 phosphorylation to activate JAK2-STAT3-CCND2 pathway. **A**–**C**. Xenograft models were established using HCT116 COP1-overexpressing and vector control cells. Mice were treated with or without oxaliplatin in each group (each group *n* = 5), and tumor growth capacity was evaluated accordingly; t-test. **D** A heatmap was generated to compare the gene expression profiles between HCT116 COP1-OE cells and HCT116 vector control cells. **E** GO and KEGG analyses were performed to identify biological processes and signaling pathways enriched among the upregulated genes in HCT116 COP1-OE cells. **F** A heatmap was generated to compare the gene expression profiles between oxaliplatin-resistant HCT116 cells and HCT116 control cells. **G** Immunoblot analysis of the COP1–DAPK3–MYL9 signaling axis in RKO and SW480 cells. Cells were transduced with COP1 or control constructs, with or without shRNA-mediated DAPK3 knockdown. Protein levels of COP1, LUZP1, MYL9, DAPK3, phosphorylated MYL9 (p-MYL9), JAK2, phosphorylated JAK2 (p-JAK2), STAT3, phosphorylated STAT3 (p-STAT3), and CCND2 were examined. **H** Xenograft models were established using COP1-OE HCT116 cells, followed by treatment with oxaliplatin and HS94. Tumor growth was then monitored to evaluate the effects of the combined treatment (each group *n* = 5); t-test. **I** Representative immunohistochemical staining of MKI67 in COP1-overexpressing tumor tissues from mice treated with placebo, oxaliplatin, or oxaliplatin combined with the DAPK3 inhibitor HS94. Right, quantification of the proportion of MKI67-positive cells. Scale bar = 100 μm. Data are presented as mean ± SD. t-test. ns, not significant; * *P* < 0.05, ** *P* < 0.01, *** *P* < 0.001, **** *P* < 0.0001

It was observed that tumors derived from COP1-OE HCT116 cells exhibited resistance to oxaliplatin (Fig. 8A and C). Following oxaliplatin treatment, tumor growth in the COP1-OE group was not significantly suppressed compared to the untreated COP1-OE group, whereas vector control tumors showed a significant growth inhibition. This supported the conclusion that elevated COP1 expression contributes to oxaliplatin resistance in vivo. In squamous cervical cancer, MYL9 has been reported to activate JAK2 phosphorylation [32]. Furthermore, phosphorylated JAK2 has been reported to activate STAT3 phosphorylation, which subsequently promoted CCND2 expression, leading to enhanced tumor stemness in CRC [33]. Given that tumor stemness was considered as a key factor contributing to oxaliplatin resistance [34], we explored whether COP1 might enhance MYL9 phosphorylation via LUZP1 degradation, potentially activating the JAK2-STAT3-CCND2 signaling axis and contributing to increased tumor stemness and oxaliplatin resistance in CRC.

To investigate transcriptional alterations induced by COP1 overexpression, we performed RNA sequencing on COP1-OE HCT116. Transcriptomic profiling revealed a noticeable upregulation of CCND2 in the COP1-overexpressing group, whereas no significant changes were observed in the expression levels of JAK family (including JAK2), STAT family (including STAT3), or MYL9 (Fig. 8D). GO and KEGG pathway enrichment analyses suggested that COP1 overexpression was primarily linked to cell proliferation and stemness-related programs, with a concurrent enrichment of platinum drug resistance pathways (Fig. 8E). We also performed RNA-seq analysis on the established oxaliplatin-resistant HCT116. Compared to the control, the resistant cell line exhibited elevated expression of MYL9, CCND2, and COP1, whereas the expression levels of the JAK (including JAK2) and STAT (including STAT3) families remained unchanged (Fig. 8F). Consistent with these transcriptomic findings, western blot validation demonstrated that COP1-OE increased the levels of p-MYL9, p-JAK2, p-STAT3, and CCND2 (Fig. 8G). Furthermore, DAPK3-knockdown based rescue experiments revealed that silencing DAPK3 effectively abrogated COP1-induced upregulation of CCND2 expression (Fig. 8G). Conversely, COP1 knockdown resulted in the opposite effects (Figure S7). In agreement, COP1 dose-escalation experiments revealed a progressive reduction in LUZP1 accompanied by increased CCND2 expression (Figure S5). Additionally, treatment with DAPK3 inhibitor HS94 sensitized COP1-OE tumors to oxaliplatin (Fig. 8H). IHC staining demonstrated increased MKI67-positive cells in COP1-overexpressing tumors, which was significantly attenuated by HS94 treatment (Fig. 8I). Sphere formation assays showed that COP1 and CCND2 increased the size of tumor spheres (Figure S8A-S8B). Apoptosis assays demonstrated that knockdown of COP1 or CCND2 significantly increased the apoptosis rate in oxaliplatin-resistant HCT116 (Figure S8C-S8D). These results suggested that the COP1-CCND2 axis enhances cell stemness and is potentially involved in chemoresistance mechanisms.

**Fig. 9:**
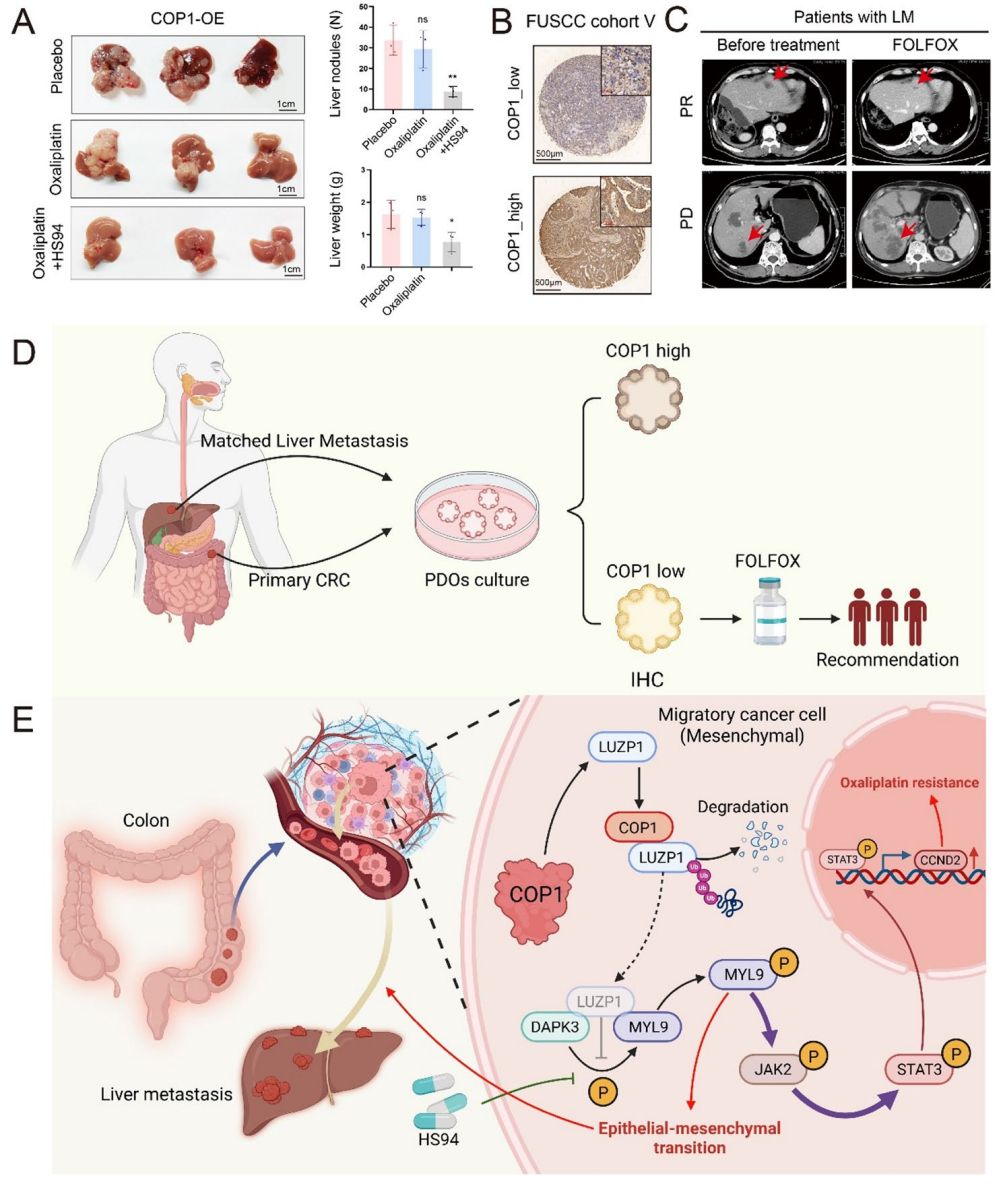
COP1 overexpression promotes resistance to oxaliplatin-based chemotherapy in colorectal cancer liver metastases (CRLM). Clinical data further indicate that elevated COP1 levels are associated with resistance to FOLFOX treatment. **A**. Representative anatomical images of the liver show morphological changes in the CRLM liver metastasis model following different drug treatments (each group *n* = 3); Data are presented as mean ± SD; t-test. **B**–**C** IHC staining was used to assess COP1 expression levels in patients treated with oxaliplatin-based FOLFOX chemotherapy (FUSCC Cohort V). MRI data showed tumor progression (PD) in patients with high COP1 expression, whereas those with low COP1 expression exhibited tumor regression (PR). **D** A schematic diagram illustrates those patients with low COP1 expression may benefit from standard FOLFOX therapy, while those with high COP1 expression may require alternative or combination treatment strategies to overcome chemoresistance. **E** Overview mechanism diagram. ns, not significant; * *P* < 0.05, ** *P* < 0.01

Moreover, in liver metastasis mouse models, we observed that COP1-OE conferred resistance to oxaliplatin in liver metastasis lesions, and this phenotype could be reversed by HS94 (Fig. 9A). We then introduced a new cohort from FUSCC (designated FUSCC Cohort V), a CRLM-TMA from patients treated with oxaliplatin-based preoperative (neoadjuvant) chemotherapy regimens (Fig. 9B). Considering that COP1 also contributed to resistance to 5-FU, we further examined the association between COP1 expression and treatment response in metastatic lesions from 26 CRLM patients treated with FOLFOX. The clinical characteristics of patients in PR and PD groups were shown in Table 4. Compared with the PR group, patients in the PD group tended to have higher T/N staging and showed a greater proportion of lymphovascular invasion, perineural invasion and KRAS mutation, although these differences did not reach statistical significance. After constructing Cox PH models and performing corrections based on Schoenfeld tests, stratified multivariable Cox regression analyses (Table 5) demonstrated that, among CRLM patients treated with neoadjuvant FOLFOX, high COP1 expression remained significantly associated with poorer overall survival (OS; *P* = 0.00045, by stratified multivariable Cox regression) and disease-free survival (DFS; *P* = 0.0091, by stratified multivariable Cox regression).

**Table 4:** Clinicopathological characteristics of FUSCC Cohort V patients with CRLM receiving neoadjuvant FOLFOX therapy (*n* = 26), stratified by response

Characteristics	*N*	FOLFOX		*P* value
		PD	PR	
Gender, n (%)				0.6680
Male	19	11(68.8%)	8(80.0%)	
Female	7	5(31.2%)	2(20.0%)	
Age, n (%)				> 0.9999
≤ 60	13	8(50.0%)	5(50.0%)	
> 60	13	8(50.0%)	5(50.0%)	
Primary site, n (%)				> 0.9999
Colon	20	12(75.0%)	8(80.0%)	
Rectum	6	4(25.0%)	2(20.0%)	
Histological type, n (%)				0.2615
Adenocarcinoma	23	13(81.3%)	10(100.0%)	
Mucinous/SRCC	3	3(18.7%)	0(0.0%)	
pT stage, n (%)				0.3853
T1/T2	1	0(0.0%)	1(10.0%)	
T3	21	13(81.3%)	8(80.0%)	
T4	4	3(18.7%)	1(10.0%)	
pN stage, n (%)				0.1504
N0	9	6(37.5%)	3(30.0%)	
N1	10	4(25.0%)	6(60.0%)	
N2	7	6(37.5%)	1(10.0%)	
Lymphovascular invasion				0.4201
Yes	13	9(56.3%)	4(40.0%)	
No	13	7(43.7%)	6(60.0%)	
Perineural invasion				0.4201
Yes	13	9(56.3%)	4(40.0%)	
No	13	7(43.7%)	6(60.0%)	
MSI station				> 0.9999
Yes	2	1(6.3%)	1(10.0%)	
No	24	15(93.7%)	9(90.0%)	
KRAS mutation				0.3004
Yes	15	11(68.8%)	4(40.0%)	
No	11	5(31.2%)	6(60.0%)	
BRAF^V600E^ mutation				0.1068
Yes	5	1 (6.3%)	4 (40.0%)	
No	21	15 (93.7%)	6 (60.0%)	

**Table 5:** Multivariate Cox regression analysis of COP1 expression and OS/DFS in CRLM-TMA with neoadjuvant FOLFOX therapy (FUSCC V)

Characteristics	Overall survival		Disease free survival	
	HR (95%CI)	*P*	HR (95%CI)	*P*
Age	1.01 (0.20, 5.18)	0.99	0.58 (0.14, 2.37)	0.44
Gender	0.12 (0.017, 0.89)	0.04	0.81 (0.17, 3.94)	0.79
Differentiation, n(%)	3.22 (0.47, 23.67)	0.23	1.72 (0.30, 9.72)	0.54
Histological type, n (%)	3.39 (0.21, 55.98)	0.39	/	/
pT stage, n (%)	0.076 (0.004, 1.44)	0.086	0.079 (0.011, 0.56)	0.12
pN stage, n (%)	0.63 (0.17, 2.29)	0.49	2.10 (0.65, 6.85)	0.22
LM size, n (%)	2.05 (1.08, 3.88)	0.028	1.12 (0.71, 1.75)	0.63
LM nodule, n (%)	1.10 (0.81, 1.50)	0.53	1.32 (0.99, 1.75)	0.056
CEA, n (%)	1.49 (0.21, 10.73)	0.69	1.14 (0.22, 5.98)	0.87
MSS status, n (%)	1.48 (0.0006, 3882.93)	0.92	/	/
KRAS mutation, n(%)	8.86 (0.82,95.80)	0.072	0.27 (0.028, 2.63)	0.26
BRAF^V600E^ mutation, n(%)	11.39 (1.02, 126.22)	0.048	0.23 (0.015, 3.52)	0.29
COP1 expression	3.60 (1.76, 7.34)	0.00045	1.66 (1.13, 2.42)	0.0091

MRI revealed that after FOLFOX treatment, patients with low COP1 expression exhibited significant shrinkage of LM lesions (PR) (Fig. 9C). Further statistical analysis showed that a higher proportion of patients in the PR group had low COP1 expression (Figure S8E). We also analyzed the GEO cohort GSE69657 [35, 36] into our analysis, in which patients were treated with 5-FU, leucovorin, and oxaliplatin (FOLFOX4) chemotherapy. COP1 expression was generally higher in non-responders than in responders (Figure S8F). Although this difference did not reach statistical significance by t-test (*P* > 0.05), likely due to the limited sample size, the observed trend was consistent with our findings in the FUSCC Cohort V. Organoid assays demonstrated that P1-derived organoids, characterized by high expression of COP1 and CCND2, were more resistant to FOLFOX treatment compared to P2-derived organoids with low COP1 and CCND2 expression. Notably, knockdown of either COP1 or CCND2 markedly increased apoptosis in P1 organoids, which exhibited a high IC50 value of 32.81µM for FOLFOX (Figure S8G-S8I). Collectively, clinical response assessments together with in vivo and in vitro experiments demonstrated that elevated COP1 expression was associated with the development and enhancement of FOLFOX resistance in CRLM.

## Discussion

In this study, we uncovered a COP1-LUZP1-MYL9 signaling axis that drives CRLM progression and confers resistance to chemotherapy, particularly oxaliplatin. We demonstrated that COP1, an E3 ubiquitin ligase [37, 38], was significantly upregulated in liver metastases compared to primary tumors, and its overexpression was associated with poor prognosis, extensive tumor dissemination (liver and lymph node metastasis), and chemoresistance. Integrating multi-omics analyses, functional assays, and preclinical models, we revealed that COP1 promoted tumor progression by ubiquitinating and degrading LUZP1, thereby relieving LUZP1-mediated suppression of DAPK3 and enhancing MYL9 phosphorylation. This cascade activates the EMT pathway and the JAK2-STAT3-CCND2 signaling axis, molecular features that are commonly linked to liver metastasis and resistance to chemotherapy (Fig. 9E).

Recent studies have increasingly underscored the oncogenic potential of COP1 across a wide range of human malignancies [39]. COP1 was frequently overexpressed in cancers such as leukemia, lymphoma, melanoma, glioma, breast, lung, colorectal, renal, hepatic, ovarian, pancreatic, gastric, and bladder cancers, as demonstrated by transcriptomic analyses and immunohistochemical studies [16, 40–42]. However, most investigations to date have primarily focused on its role in primary tumors, particularly emphasizing its function in promoting cell proliferation [43, 44]. Functional experiments have shown that COP1 overexpression enhanced tumor cell proliferation [45], colony formation, and in vivo tumor growth, whereas siRNA-mediated knockdown of COP1 markedly suppressed tumor progression and induced apoptosis in models including hepatocellular carcinoma, glioma, and chronic lymphocytic leukemia [46]. In our previous work, we further demonstrated that the CUL4B-DDB1-COP1 complex facilitated CRC progression by promoting the ubiquitin-mediated degradation of UTX, thereby accelerating CRC cell proliferation [18].

Despite its established role in primary tumor growth, the function of COP1 in cancer metastasis—particularly in CRLM—remained poorly understood. To address this gap, our study identified COP1 as a pivotal regulator of CRLM. Elevated COP1 expression in liver metastatic lesions was consistently observed across multiple cohorts, including PDOs, TMA, and scRNA sequencing datasets. As a RING finger-type E3 ubiquitin ligase, COP1 maintains protein homeostasis by mediating substrate-specific ubiquitination and degradation. Given its canonical function, we hypothesized that COP1 may promote CRLM through its E3 ligase activity. To elucidate the downstream mechanisms underlying COP1-driven CRLM progression, we identified LUZP1 as a key substrate of COP1. Mechanistically, we showed that COP1 bound to LUZP1 via its WD40 domain and promoted its ubiquitination and degradation through the RING domain. Notably, LUZP1 has been previously reported to inhibit DAPK3-mediated phosphorylation of MYL9, a well-established regulator of cell motility [31]. We therefore proposed that COP1-mediated degradation of LUZP1 would relieve its inhibitory effect on DAPK3, leading to enhanced MYL9 phosphorylation and activation of the EMT pathway. This mechanistic axis was further supported by reciprocal alterations in EMT markers—including E-cadherin, N-cadherin, and Vimentin—following modulation of COP1 or LUZP1 expression.

Oxaliplatin, 5-FU and irinotecan represent first-line chemotherapeutic agents for CRLM [47–49]. However, a considerable proportion of CRLM patients exhibit poor therapeutic responses, reflecting the major challenge of intrinsic or acquired chemoresistance [50]. Resistance to these cornerstone agents frequently arises during treatment, contributing to tumor progression, recurrence, and adverse clinical outcomes [51]. Therefore, elucidating the molecular mechanisms underlying oxaliplatin, 5-FU or irinotecan resistance and developing strategies to restore chemosensitivity remain urgent priorities. Having identified COP1 as a critical regulator of CRLM progression, we investigated its role in chemotherapy resistance. Given that enhanced tumor cell proliferation is a well-recognized mechanism of chemoresistance [52], and COP1 is known to promote tumor growth [16], we hypothesized that COP1 might also play a role in mediating resistance to chemotherapy. Consistent with this, our drug sensitivity profiling revealed a strong positive correlation between COP1 expression and oxaliplatin resistance. PDOs and CRC cell lines with COP1 overexpression displayed diminished sensitivity to oxaliplatin, whereas COP1 knockdown significantly restored drug responsiveness. Notably, COP1 overexpression also conferred a modest increase in resistance to SN-38, resembling its effect on 5-FU (Figure S6D-S6E). These findings suggest that COP1 promotes a broad, multidrug-resistant phenotype rather than conferring resistance exclusive to oxaliplatin. Mechanistically, the proposed COP1-LUZP1-DAPK3-MYL9 axis could establish such a generalized survival state. By inducing LUZP1 degradation and activating DAPK3, COP1 enhances MYL9 phosphorylation, thereby activating the JAK2-STAT3-CCND2 signaling cascade. This pathway drives cell survival and anti-apoptotic programs and sustains cancer stem-like properties, both of which are linked to therapy resistance. Concurrently, activation of the COP1-LUZP1-DAPK3-MYL9 axis promotes epithelial-mesenchymal transition, a hallmark of adaptive resistance. Thus, COP1 overexpression rewires cellular fate toward a pro-survival, anti-apoptotic, and stemness-associated state. Although the specific effectors for different drugs may vary, activation of this core program likely establishes a shared, therapy-tolerant state, explaining the observed cross-resistance. Collectively, these findings indicate that COP1 contributes to CRLM progression and chemotherapy resistance through the COP1-LUZP1-DAPK3-MYL9 signaling cascade.

Several E3 ubiquitin ligases have been implicated in colorectal cancer progression and therapeutic resistance. Among them, FBXW7 is a representative example whose tumor-suppressive function is primarily mediated through the canonical Cullin-RING ligase (CRL) machinery, more specifically the SCF complex composed of SKP1, CUL1, an F-box protein, and RBX1, which belongs to the CRL1 family of E3 ubiquitin ligases [53]. By promoting the ubiquitin-dependent degradation of oncogenic substrates such as ZEB2 and Mcl-1, FBXW7 suppresses tumor metastasis and therapy resistance [54, 55]. Similarly, Smurf2, a HECT-type E3 ligase, functions as a tumor suppressor in CRC by degrading pro-metastatic and pro-glycolytic substrates such as RhoA and ChREBP, thereby inhibiting cancer cell migration and metabolic reprogramming [56, 57]. These examples illustrate that many E3 ligases act as tumor suppressors in CRC, restraining malignancy through targeted degradation of oncoproteins. In contrast, our findings reveal that COP1 functions as a pro-tumorigenic E3 ligase in colorectal cancer liver metastasis and oxaliplatin resistance. Unlike FBXW7 or Smurf2, which rely on canonical CRL or HECT-type catalytic mechanisms, COP1 mediates the degradation of its substrate LUZP1 through a CRL-independent mode. Although COP1 has previously been linked to CRL-associated functions, our data demonstrate that COP1 directly ubiquitinates and degrades LUZP1 in a fully CRL-independent manner. This unique regulatory mode distinguishes COP1 from all previously characterized E3 ligases in CRC. Together, these findings highlight an unrecognized oncogenic function of COP1 and significantly expand the mechanistic diversity of E3 ligase signaling in CRC metastasis and chemoresistance.

The translational relevance of our findings is further supported by integrated analyses of clinical cohorts and PDO models. Functionally, we demonstrated that knockdown of COP1 or its downstream effector CCND2 in PDOs with high COP1 expression restored sensitivity to FOLFOX, a first-line chemotherapeutic regimen. These results suggest a potential precision medicine framework in which postoperative tumor specimens from CRC patients could be used to establish PDOs for COP1 expression profiling, thereby informing individualized therapeutic strategies. Specifically, patients with low COP1 expression may benefit from standard FOLFOX therapy, whereas those with high COP1 expression might require alternative or combination treatment approaches to overcome chemoresistance (Fig. 9D).

By explicitly comparing our findings with prior studies, we further strengthened the translational framing. Previous organoid-based investigations have largely focused on phenotypic drug screening without mechanistic resolution. In contrast, our work leverages PDOs as a discovery platform followed by integrated mechanistic dissection and in vivo validation, thereby systematically establishing the functional relevance of the COP1-LUZP1-DAPK3-MYL9 axis. Although DAPK3 has been implicated in cytoskeletal dynamics, its upstream regulation and contribution to CRC metastasis and chemoresistance had not been previously elucidated. Thus, our study not only highlights a distinct mechanistic role of COP1 but also delineates a previously unrecognized signaling cascade with potential translational implications for inhibiting metastasis and therapy resistance in colorectal cancer.

While our study provided comprehensive mechanistic insights, certain limitations warrant discussion. First, owing to the technical challenges associated with the long-term maintenance of paired CRLM PDOs, the number of paired PDO samples included was relatively limited. Nevertheless, by integrating paired CRLM tissue datasets from public repositories and liver metastasis–derived PDX models, we confirmed the trend of elevated COP1 expression in metastatic lesions, supporting the robustness and broader applicability of our observations. Future studies incorporating larger patient cohorts and expanded PDO collections will be necessary to increase statistical power and further validate these conclusions.

Second, although our mechanistic and functional experiments support the proposed COP1-LUZP1-MYL9 model, further research is needed to establish a direct and linear causal relationship in human patients. Moreover, while PDOs represent a powerful platform for mechanistic interrogation, they do not fully recapitulate key in vivo complexities, including the dynamic tumor microenvironment, stromal and immune interactions, and systemic pharmacokinetic and pharmacodynamic influences, all of which critically shape therapeutic response and delineate the boundary between mechanistic insight and direct clinical predictability. Therefore, our PDO experiments should be viewed primarily as a discovery platform for biologically relevant resistance pathways rather than as an immediate clinical decision-making tool. To bridge this translational gap, future efforts integrating PDOs with immune-competent model systems, incorporating pharmacokinetic considerations, and pursuing prospective validation in clinical cohorts or organoid-guided therapeutic pipelines will be important.

Third, the precise structural determinants underlying the COP1-LUZP1 interaction remain to be fully defined and will require detailed crystallographic or structural analyses. Such information would be essential for the rational design of strategies to modulate COP1 function, as direct pharmacological inhibition of E3 ubiquitin ligases remains technically challenging. Potential alternative approaches—such as disrupting specific COP1-substrate interactions or leveraging PROTACs—may represent more feasible avenues. Moreover, HS94 was used in this study primarily as a tool compound to interrogate DAPK3 biology. Its potential for clinical translation will therefore require comprehensive evaluation of its pharmacokinetic properties, as well as its safety and toxicity profiles. Thus, rather than proposing immediate therapeutic deployment, our findings highlight the COP1-LUZP1-DAPK3-MYL9 axis as a mechanistic vulnerability that may inform future development of rational combination strategies, including sensitization to oxaliplatin-based chemotherapy.

## Conclusion

In summary, this study identifies the COP1-LUZP1-MYL9 axis as a key mechanistic pathway associated with CRLM and oxaliplatin-based chemoresistance. By promoting LUZP1 degradation, COP1 activates the DAPK3-MYL9 signaling axis, leading to EMT-related features and the chemoresistance-associated JAK2-STAT3-CCND2 pathway. Our work not only advances the molecular understanding of CRC metastasis but also identifies COP1 as a promising therapeutic target. Future efforts to translate these findings into clinical practice could significantly improve outcomes for patients with CRLM.

## Supplementary Information

Below is the link to the electronic supplementary material.

Supplementary Table S1

Supplementary Table S4

Supplementary Table S3

Supplementary Table S2

Supplementary Material 1

## Data Availability

The transcriptome expression data of cell lines in this study are listed in Supplementary Table S2. Single-cell RNA-sequencing and mRNA expression raw data of PDOs are available at the National Genomics Data Center (NGDC) under the accession number HRA016322.

## Abbreviations

CRC: Colorectal cancer
CRLM: Colorectal cancer liver metastasis
LM: Liver metastasis
PDO: Patient-derived organoid
OS: Overall survival
DFS: Disease-free survival
IHC: Immunohistochemistry
CHX: Cycloheximide
Co-IP: Co-immunoprecipitation
scRNA: Single-cell RNA
RNA-seq: RNA sequencing
WES: Whole exome sequencing
5-FU: 5-Fluorouracil
EMT: Epithelial-mesenchymal transition
PR: Partial response
SD: Stable disease
PD: Progressive disease
