# Supplementary material for: Multi-omics analysis of patient-derived organoids reveals that E3 ligase COP1 promotes liver metastasis and oxaliplatin resistance in colorectal cancer through LUZP1 degradation and MYL9 phosphorylation
Source: Exp Hematol Oncol. 2026 Apr 5;15:43. doi: 10.1186/s40164-026-00771-7 (PMC13094177; doi:10.1186/s40164-026-00771-7)
Supplement: Supplementary file 5 — Supplementary Material 1 [file 40164_2026_771_MOESM17_ESM.docx]

**Multi-Omics Analysis of Patient-Derived Organoids Reveals that E3 Ligase COP1 Promotes Liver Metastasis and Oxaliplatin Resistance in Colorectal Cancer through LUZP1 Degradation and MYL9 Phosphorylation**

*Running title*: COP1 Promotes CRC Liver Metastasis and Chemoresistance

Ruijia Zhang^1,2†^, Wenqin Luo^1,2†^, Qikai Zhou^3†^, Dongguo Liang^4†^, Yuankai Hao^3^, Fan Chen^1,2^, Yulin Qiu^1,2^, Yixian Cao^3^, Zezhi Shan^1,2^, Yu Zhang^1,2^, Qingguo Li^1,2^, Sanjun Cai^1,2^, Dakui Luo^1,2*^, Shaobo Mo^1,2*^, Bin Ma^5*^, Xinxiang Li^1,2*^

^1^ Department of Colorectal Surgery, Fudan University Shanghai Cancer Center, Shanghai, 200032, China.

^2^ Department of Oncology, Shanghai Medical College, Fudan University, Shanghai, 200032, China.

^3^ School of Biomedical Engineering, Med-X Research Institute, Shanghai Jiao Tong University, Shanghai, China.

^4^ Shanghai Institute of Hematology, National Research Center for Translational Medicine, State Key Laboratory of Medical Genomics, Ruijin Hospital Affiliated to Shanghai Jiao Tong University School of Medicine, Shanghai, China.

^5^ Shanghai Key Laboratory for Cancer System Regulation and Clinical Translation, Shanghai Jiading District Central Hospital, Shanghai, China.

^†^ Ruijia Zhang, Wenqin Luo, Qikai Zhou, and Dongguo Liang contributed equally to this work.

^*^ Correspondance: Xinxiang Li (xinxiangli@fudan.edu.cn), Bin Ma (bin.ma@outlook.com), Shaobo Mo (shaobom@shca.org.cn), and Dakui Luo (dkluo17@fudan.edu.cn).

**Supplementary Figures**

**Figure S1**

**
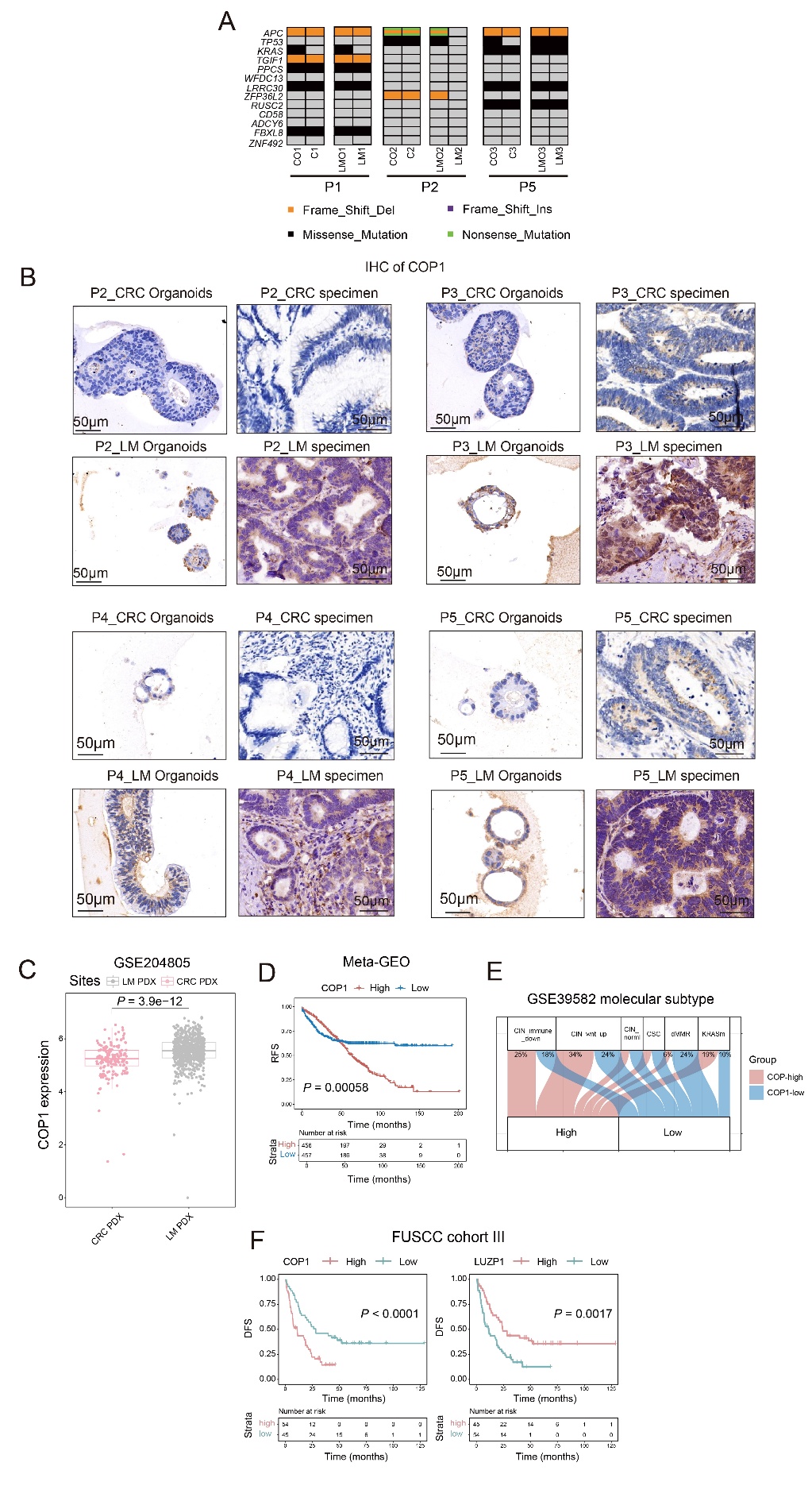
**

**Figure S1. Elevated COP1 expression in colorectal cancer liver metastases predicts poor prognosis, related to Figure 1.**

**A.** Waterfall plot showing somatic mutation profiles of representative CRC primary tumors and matched LM from three patients (P1, P2, and P5). Mutation types are indicated by color, including missense mutations, frameshift insertions/deletions, and nonsense mutations. **B.** Representative IHC staining of COP1 in PDOs established from primary CRC tissues and matched liver metastases (LM) (patients P2-P5), together with the corresponding source specimen sections. Scale bar = 50μm. **C.** Comparison of COP1 expression between CRC-derived and LM-derived patient-derived xenografts (PDXs) in the GSE204805 dataset. *P* value was calculated using t-test. **D.** Kaplan-Meier analysis of recurrence-free survival (RFS) in a meta-GEO cohort, stratified by COP1 expression (high vs low). *P* value was determined by the log-rank test. **E.** Sankey diagram showing the distribution of molecular subtypes (GSE39582) in COP1-high and COP1-low CRC tumors, indicating the association between COP1 expression and distinct molecular subgroups. **F.** Kaplan-Meier analysis of disease-free survival (DFS) in the FUSCC cohort III, stratified by COP1 (left) or LUZP1 (right) expression levels. *P* values were calculated using the log-rank test.

**Figure S2**

**
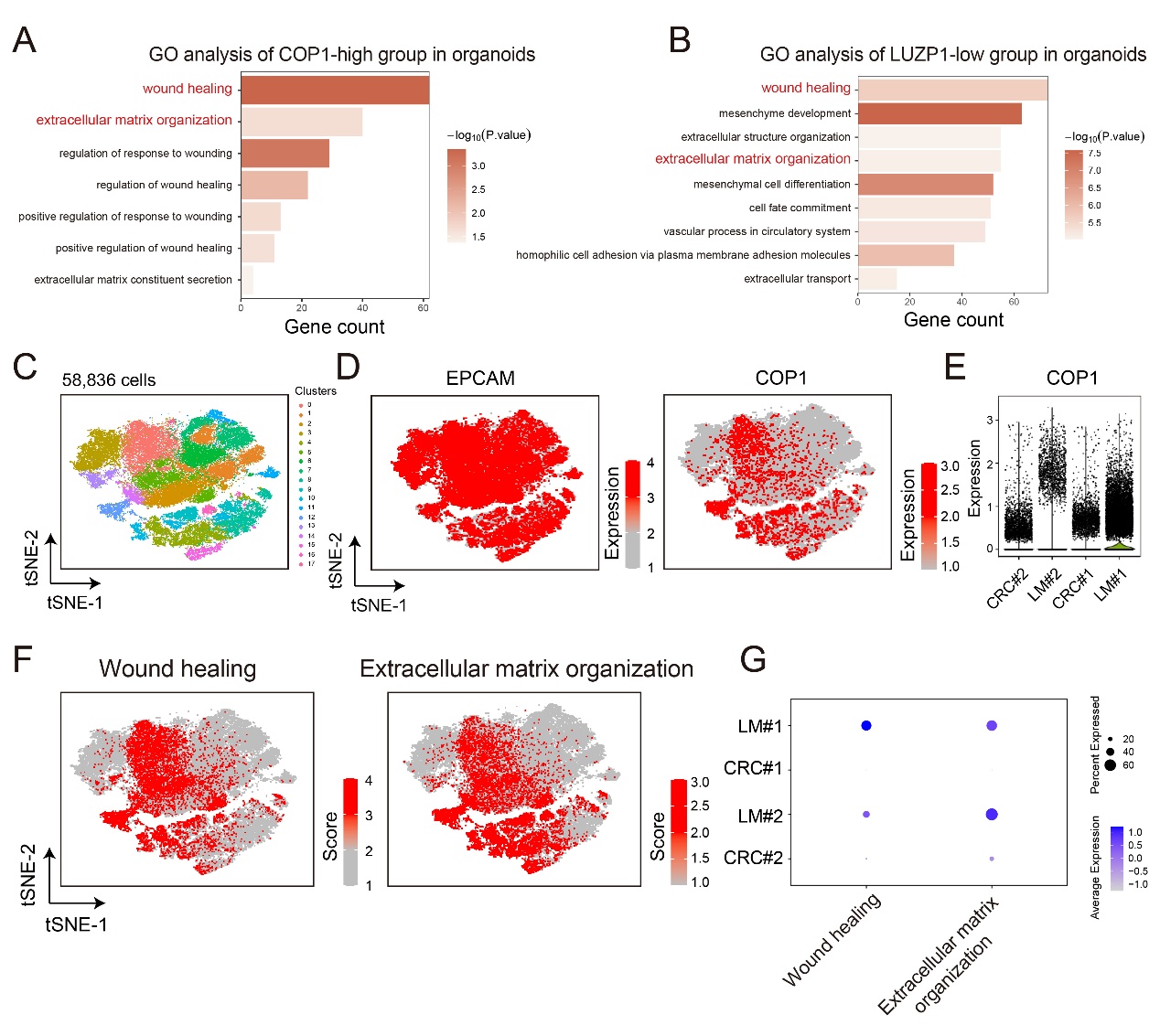
**

**Figure S2. COP1- and LUZP1-associated transcriptional programs and single-cell characteristics in colorectal cancer organoids.**

**A-B.** Cells were stratified based on the median single-cell expression levels of COP1 or LUZP1. GO enrichment analysis was then performed on cells with high COP1 expression or on high LUZP1 expression to identify associated biological processes between the two groups. **C.** t-SNE visualization of 58,836 single cells derived from CRLM PDO samples, colored by cluster identity. **D.** Feature plots showing expression of the epithelial marker EPCAM (left) and COP1 (right) projected onto the t-SNE embedding. **E.** Violin plot showing COP1 expression levels across CRC- and LM–derived organoid samples. **F.** Single-cell gene set scoring visualized on t-SNE plots for wound healing–related and extracellular matrix organization–related gene signatures score. **G.** Dot plot summarizing the average expression and percentage of cells expressing wound healing and extracellular matrix organization gene signatures in CRC- and LM-derived organoids.

**Figure S3**


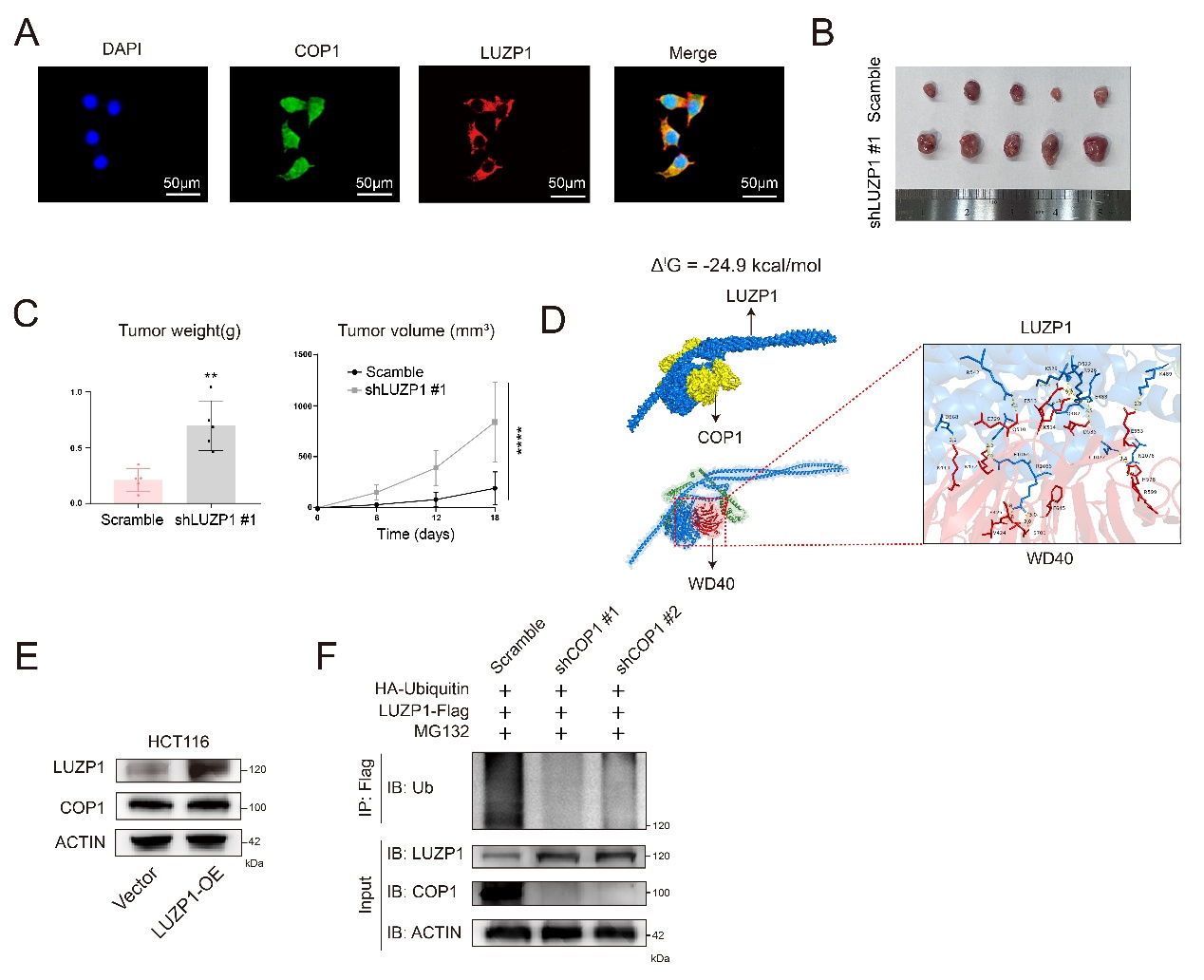


**Figure S3.** **COP1 interacts with LUZP1 and promotes its ubiquitin-mediated degradation.**

**A.** Immunofluorescence staining showing the subcellular localization and partial colocalization of COP1 (green) and LUZP1 (red). Nuclei were counterstained with DAPI (blue). Scale bar = 50μm. **B.** Subcutaneous xenograft assays showing enhanced tumor growth following LUZP1 knockdown. Left, representative images of excised tumors from mice injected with HCT116 cells expressing scramble control or shLUZP1 #1 (each group n = 5). Middle, tumor weights at endpoint. Right, tumor growth curves. Data are presented as mean ± SD. **D.** Protein–protein docking analysis predicted that COP1 binds LUZP1 through its WD40 domain. The modeled complex showed multiple hydrogen bonds at the COP1–LUZP1 interface, and PDBePISA analysis estimated a favorable binding free energy (ΔG = −24.9 kcal/mol), supporting a stable interaction between COP1 and LUZP1. **E.** Immunoblot analysis confirming LUZP1 knockdown and the corresponding change in COP1 protein levels in HCT116 cells. **F.** Anti-ubiquitin immunoblotting of immunoprecipitated exogenous LUZP1 in 293T cells transfected with the indicated plasmids, with or without MG132 treatment.

**Figure S4**

**
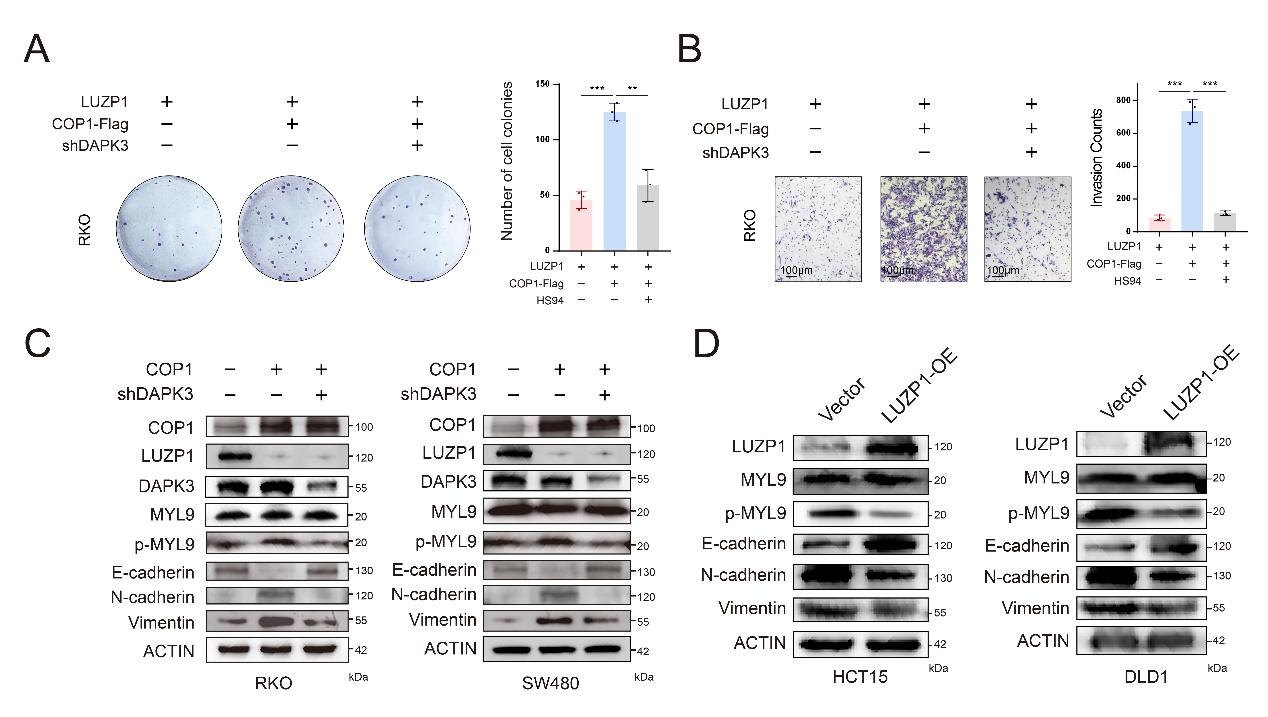
**

**Figure S4.** **COP1 promotes EMT activation by degrading LUZP1 and enhancing DAPK3-mediated MYL9 phosphorylation.**

**A.** Colony formation assays in RKO cells showing that COP1 overexpression promotes colony formation, which is partially reversed by DAPK3 knockdown. Left, representative images; right, quantification of colony numbers (each group n = 3). **B.** Transwell invasion assays demonstrating that COP1 enhances invasive capacity in RKO cells, which is attenuated by DAPK3 inhibition. Left, representative images; right, quantification of invaded cells (each group n = 3). Scale bar = 100μm. **C.** Western blot was performed following COP1-OE to assess the expression levels of downstream MYL9, phosphorylated MYL9 (p-MYL9), and EMT-related proteins. **D.** Western blot was performed following LUZP1-OE to assess the expression levels of downstream MYL9, phosphorylated MYL9 (p-MYL9), and EMT-related proteins. ** *P* < 0.01, *** *P* < 0.001, **** *P* < 0.0001.

**Figure S5**

**
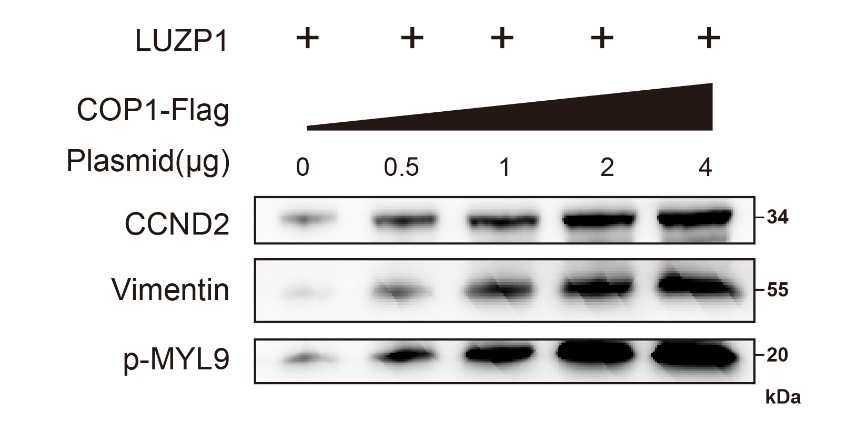
**

**Figure S5.  Immunoblot analysis showing dose-dependent effects of COP1 overexpression.**

Increasing amounts of COP1-FLAG plasmid were transfected into 293T cells in the presence of LUZP1, resulting in progressive changes in the protein levels of CCND2, vimentin, and phosphorylated MYL9 (p-MYL9), with ACTIN serving as the loading control as shown in Figure 5F.

**Figure S6**

**
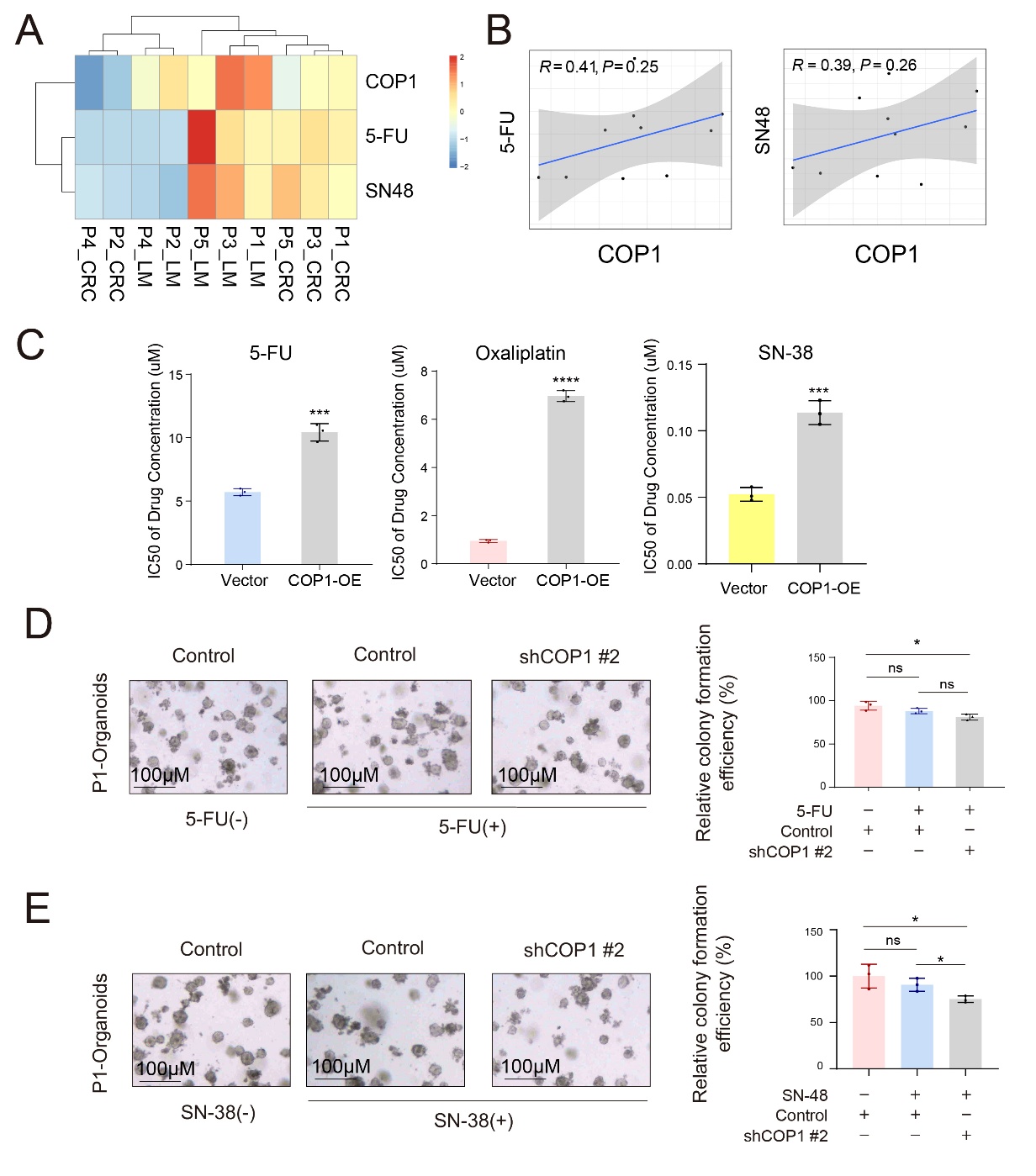
**

**Figure S6. COP1 promotes a multidrug-resistant phenotype in colorectal cancer models.**

**A.** Heatmap showing the relative IC50 values of COP1, 5-fluorouracil (5-FU), and SN-38 across PDOs from primary CRC and liver metastasis (LM). **B.** Correlation analysis between COP1 expression and IC50 values of 5-FU (left) and SN-38 (right) across PDO samples. Pearson correlation coefficients (R) and P values are indicated. **C.** IC50 values of 5-FU, oxaliplatin, and SN-38 in colorectal cancer cells expressing vector control or COP1 overexpression (COP1-OE) (each group n = 3). Data are presented as mean ± SD. Statistical significance was determined using a t-test. **D.** Representative bright-field images of P1 patient-derived organoids treated with 5-FU in scramble control or COP1-knockdown (shCOP1 #2) conditions. Right, quantification of relative colony-forming efficiency (each group n = 3). Scale bar = 100μm. **E.** Representative images of P1 patient-derived organoids treated with SN-38 under scramble control or shCOP1 #2 conditions, with quantification of relative colony-forming efficiency shown on the right (each group n = 3). Scale bar = 100μm ns, not significant; * *P* < 0.05, *** *P* < 0.001, **** *P* < 0.0001.

**Figure S7**

**
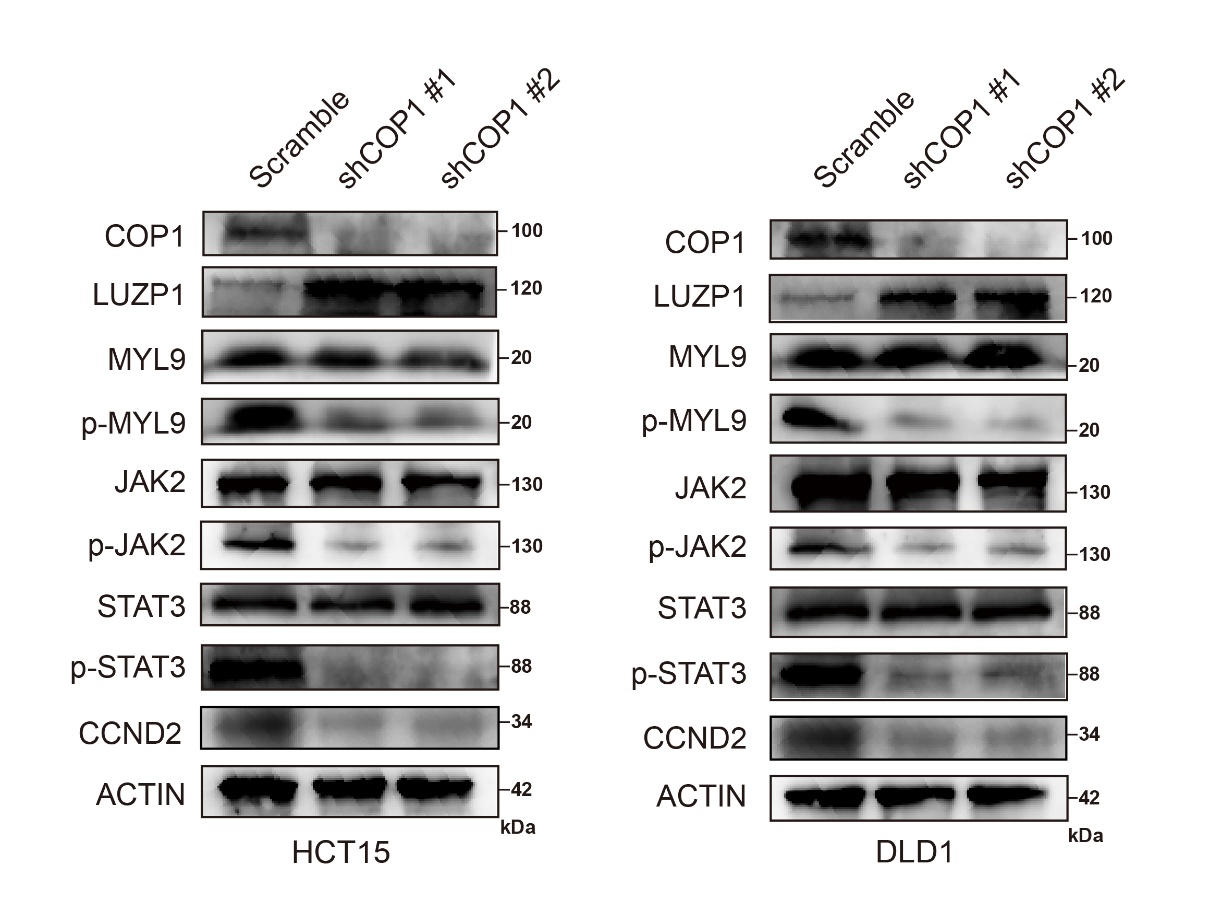
**

**Figure S7. COP1 regulates the MYL9–JAK2–STAT3–CCND2 signaling axis in colorectal cancer cells.**

Immunoblot analysis of the MYL9–JAK2–STAT3 signaling pathway in HCT15 and DLD1 cells following COP1 knockdown. Cells were transduced with scramble control or two independent COP1 knockdown constructs (shCOP1 #1 and shCOP1 #2). Protein levels of COP1, LUZP1, MYL9, phosphorylated MYL9 (p-MYL9), JAK2, phosphorylated JAK2 (p-JAK2), STAT3, phosphorylated STAT3 (p-STAT3), and CCND2 were examined.

**Figure S8**

**
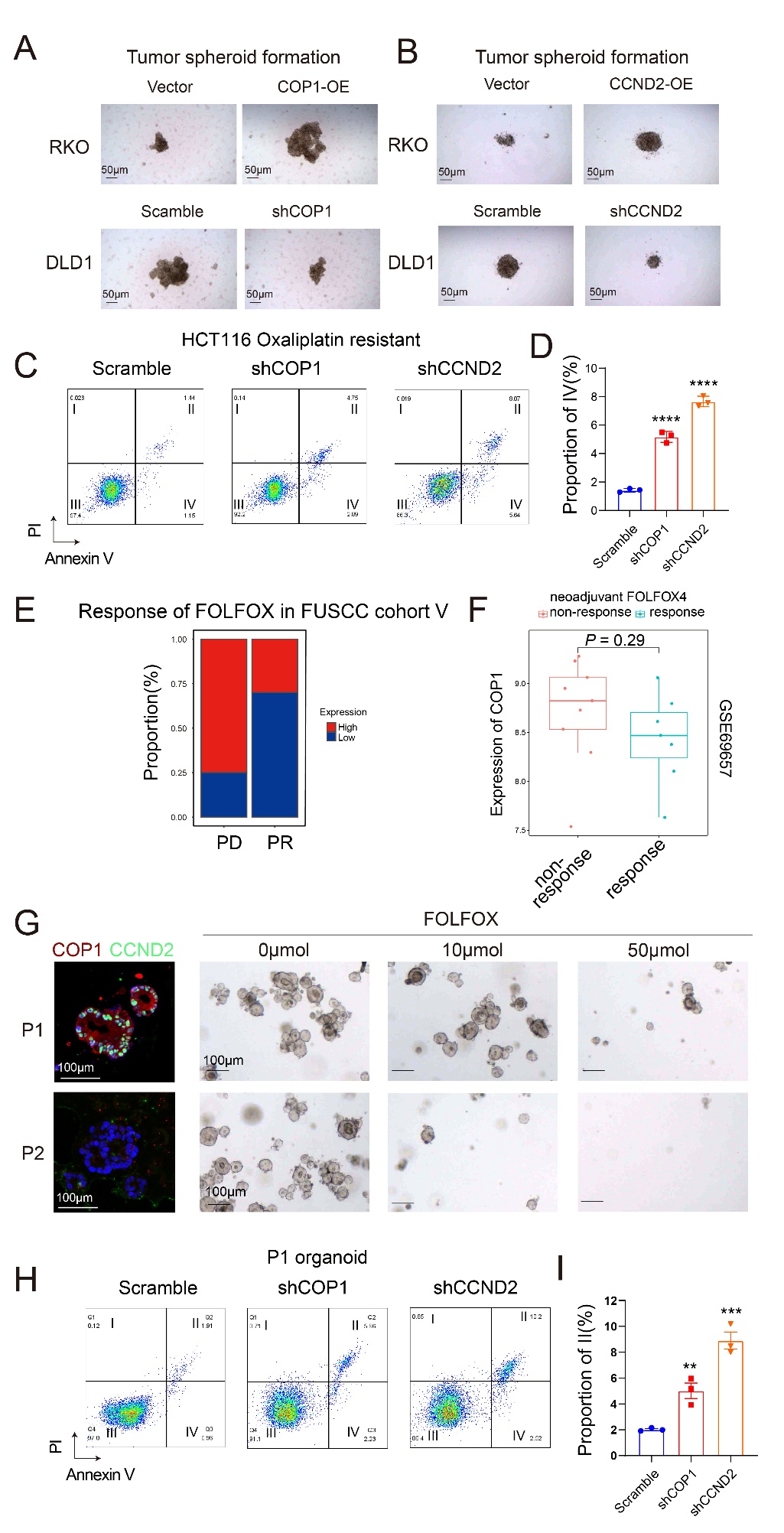
**

**Figure S8. COP1 promotes CCND2 expression, which enhances tumor stemness and contributes to chemotherapy resistance.**

**A.** Sphere formation assays were performed using COP1-overexpressing (COP1-OE) and COP1-knockdown (shCOP1 #2) cell lines to evaluate their self-renewal and tumor stemness capacities. **B.** Sphere formation assays were performed using CCND2-overexpressing (CCND2-OE) and CCND2-knockdown (shCCND2) cell lines to evaluate their self-renewal and tumor stemness capacities. **C-D.** Apoptosis assays were conducted in HCT116 oxaliplatin-resistant cells following knockdown of either COP1 or CCND2 to assess the extent of cell apoptosis (each group n = 3); t-test. **E.** Distribution of treatment responses to FOLFOX chemotherapy in the FUSCC cohort V, stratified by COP1 expression levels (high vs low). PD, progressive disease; PR, partial response. **F.** Comparison of COP1 expression between responders and non-responders to neoadjuvant FOLFOX chemotherapy in GSE69657 cohort. Data are shown as box plots with individual data points. Statistical significance was assessed using a t-test; P value is indicated. **G.** Bright-field microscopy images show the cell viability of organoids derived from Patient P1 (high COP1 and CCND2 expression) and Patient P2 (low COP1 and CCND2 expression) under different concentrations of FOLFOX treatment. **H-I.** Apoptosis assays were conducted in FOLFOX-resistant P1-derived PDOs following knockdown of either COP1 or CCND2 to assess the extent of cell apoptosis (each group n = 3); t-test. ** *P* < 0.01, *** *P* < 0.001, **** *P* < 0.0001.

**Supplementary Tables**

**Supplementary Table S1. Demographic and clinicopathological characteristics of 5 patients in FUSCC cohort I.**

*Please see separated Excel spreadsheet file.*

**Supplementary Table S2. The transcriptome expression data of cell lines in this study.**

*Please see separated Excel spreadsheet file.*

**Supplementary Table S3. Immunoprecipitation-Mass Spectrometry (IP-MS) analysis of COP1-interacting proteins.**

*Please see separated Excel spreadsheet file.*

**Supplementary Table S4. IC50 of PDOs.**

*Please see separated Excel spreadsheet file.*
